# Downregulation of ASPP2 promotes gallbladder cancer metastasis and macrophage recruitment via aPKC-ι/GLI1 pathway

**DOI:** 10.1038/s41419-018-1145-1

**Published:** 2018-11-02

**Authors:** Li Tian, Zhengdong Deng, Lei Xu, Tao Yang, Wei Yao, Lei Ji, Yun Lu, Jian Zhang, Yan Liu, Jianming Wang

**Affiliations:** 1 Department of Biliary and Pancreatic Surgery, Affiliated Tongji Hospital, Tongji Medical College, Huazhong University of Science and Technology, 430030 Wuhan, Hubei China; 2 Department of Geriatrics, Affiliated Tongji Hospital, Tongji Medical College, Huazhong University of Science and Technology, 430030 Wuhan, Hubei China

## Abstract

Gallbladder cancer (GBC) is a highly malignant bile duct cancer with poor prognosis due to early invasion and metastasis. However, the molecular mechanisms through which GBC cells interact with the tumor microenvironment (TME) remain poorly understood. Here, we examined the role of the tumor suppressor apoptosis-stimulating of p53 protein 2 (ASPP2) in regulating GBC invasion and metastasis and macrophage recruitment. The clinicopathological significance of ASPP2 expression was measured by immunohistochemical analysis in 72 patients with GBC. Lentivirus-mediated knockdown or overexpression of ASPP2 was used to investigate the biological functions and molecular mechanisms of ASPP2 in GBC cells. Our data showed that downregulation of ASPP2 in patients with GBC was linked to poor prognosis. Knockdown of ASPP2 induced epithelial–mesenchymal transition (EMT) in GBC cells and influenced the TME. Mechanistically, we further confirmed that ASPP2 affected the expression and protein binding between atypical protein kinase C (aPKC)-ι and glioma-associated oncogene homolog 1 (GLI1). ASPP2 also induced C−C motif chemokine ligand (CCL) 2, CCL5, and tumor necrosis factor-α secretion by cancer cells, thereby promoting macrophage recruitment. The latter also induced EMT-like changes in GBC. Furthermore, ASPP2 deficiency regulated GLI1 transcriptional activity via the noncanonical Hedgehog (Hh) pathway and aPKC-ι/GLI1 signaling loop and promoted GLI1 nuclear localization and binding to the promoters of target genes. Our findings revealed that downregulation of ASPP2 promoted GBC invasion and metastasis through the aPKC-ι/GLI1 pathway and enhanced macrophage recruitment. Thus, ASPP2/aPKC-ι/GLI1 pathway may be a potential therapeutic target for the treatment of GBC.

## Introduction

Gallbladder cancer (GBC), a primary malignancy of the biliary tract, is the sixth most common gastrointestinal cancer and has a 5-year survival rate of 5%^1,2^. Such poor prognosis is due, in part, to its aberrant anatomical features, aggressive biological behaviors, and lack of sensitive screening tests for early diagnosis, resulting in loss of the opportunity for early treatment^1,3^. Although radical resection is the most promising potential curative approach for patients, less than 10% of patients are considered candidates for resection because of advanced stage disease, and nearly 50% of patients exhibit lymph node metastasis on initial diagnosis^4,5^.

Metastasis is a highly complex biological process involving a multistep cascade of genetic and epigenetic events. For tumors to metastasize, the cancer cells must obtain enhanced invasive capacity, and the tumor microenvironment (TME) must be remodeled^6^. Growing evidence has supported the concept that the epithelial-to-mesenchymal transition (EMT) plays pleiotropic roles in tumor metastasis^7,8^. We previously reported that atypical protein kinase C (aPKC)-ι, as an oncogene and key polarization regulator, is positively correlated with cholangiocarcinoma (CCA) differentiation and invasion^9^. We also showed that aPKC-ι induced the EMT in CCA cells and stimulates immunosuppression associated with Snail^10^. However, it is unknown how GBC cells modulate the TME and what the molecular mechanisms are associated with the interaction between tumor and host cells during the EMT.

Apoptosis-stimulating of p53 protein 2 (ASPP2), a haploinsufficient tumor suppressor that was originally identified as an activator of the p53 family, is a member of the ASPP family, together with ASPP1 and iASPP, and has several shared structural features, including ankyrin repeats, an SH3 domain, and a proline-rich region^11,12^. Downregulation of ASPP2 is associated with the advanced stages of many human cancers, such as breast cancer, hepatocellular carcinoma, and pancreatic cancer^13–16^. In the nucleus, direct binding with p53 and stimulation of the transactivation of p53 are downstream events of ASPP2-induced apoptosis^17^. However, clinical studies have also detected ASPP2 in the cytoplasm of cancer cells^18^. Recent studies have shown that ASPP2 controls cell polarity during central nervous system development and is colocalized with the Par3 complex to act as a regulator of cell−cell adhesion^19^. Of note, ASPP2 deficiency promoted EMT and tumor metastasis in multiple types of cancer^13^; however, it remains unknown whether ASPP2 is involved in the regulation of EMT in GBC.

Recent studies of the Hedgehog (Hh) pathway have shown that this pathway is a critical regulator of cancer progression and has fundamental roles in the development and differentiation of tissues and organs during embryonic life^20^. Aberrant activation of the Hh pathway results in a wide variety of human cancers, including GBC^21^. The transcription factor glioma-associated oncogene homolog 1 (GLI1), which is a central player in the Hh pathway, mediates Hh signaling and acts as a marker of Hh signaling activation by translocation to the nucleus^22^. Activated GLI proteins translocate into the nucleus and stimulate the transcription of Hh pathway target genes, including GLI1, protein patched homolog 1 (PTCH1), smoothened (SMO), and many survival-promoting molecules^23^.

In addition to being activated by the Hh ligand/PTCH1/SMO axis, also known as the canonical Hh pathway, a growing body of evidence suggests that activation of GLI1 in some cancers is not controlled exclusively by Hh signaling but is also modulated by other pathways, such as AKT, MAPK/ERK, and KRAS pathways in an Hh ligand/PTCH1/SMO axis-independent manner, also known as noncanonical Hh signaling^24^. Although the canonical Hh pathway has been extensively studied, it is still unclear how GLI1 is regulated through noncanonical Hh signaling. Therefore, it is important to identify other upstream regulators of GLI1.

In this study, we evaluated the fundamental roles of ASPP2 in tumor progression and the TME via the noncanonical Hh pathway involving aPKC-ι/GLI1 signaling in GBC.

## Results

### ASPP2 was downregulated and correlated with poor prognosis in patients with GBC

**Fig. 1:**
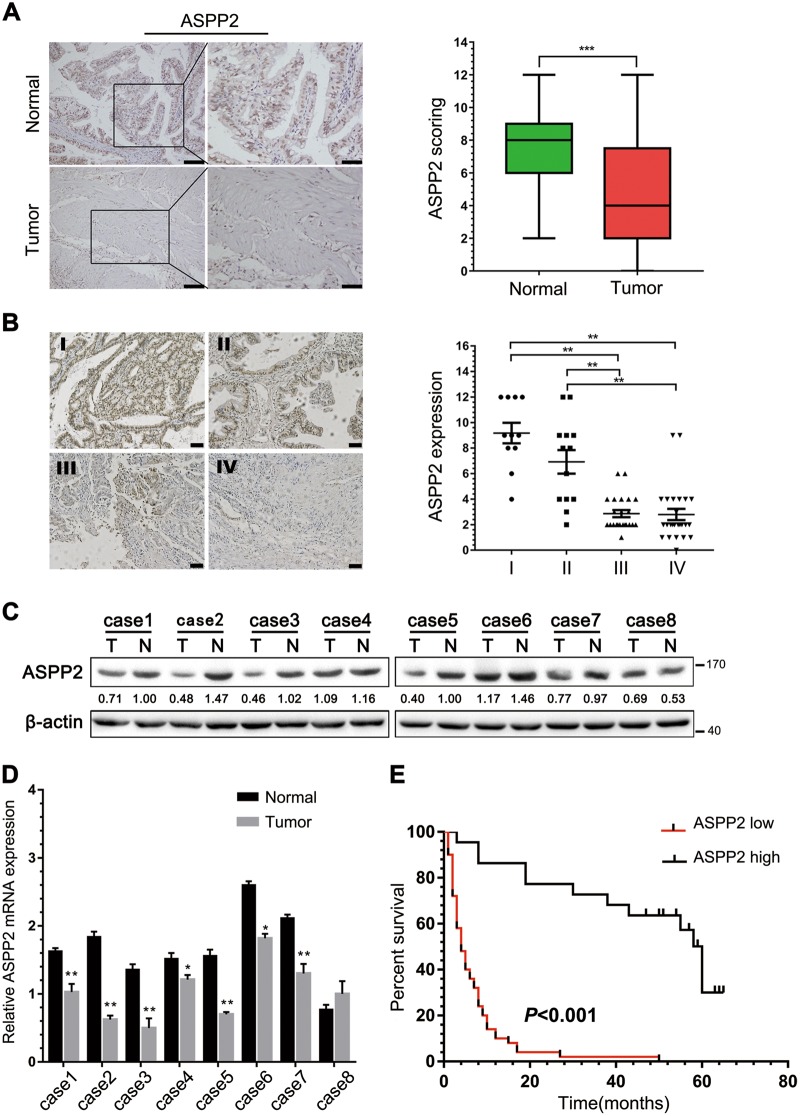
The expression and clinical significance of ASPP2 in GBCs. **a** Left panel, representative images of IHC staining of ASPP2 expression in 72 human GBC tissues and pair-matched normal gallbladder tissues. Scale bar, 100 μm (left) or 50 μm (right). Right panel, box plot showing the statistical analysis of ASPP2 expression in GBC samples. **b** IHC staining for detection of ASPP2 in 72 human GBC samples of different clinical stages (I, II, III, and IV) according to the seventh AJCC staging criteria (left) and scatter plot showing the ASPP2 expression level in each group (right). Scale bar, 50 μm. **c**, **d** ASPP2 protein and mRNA expression in representative GBC samples (T) and pair-matched normal tissues (N) evaluated by western blotting and qPCR, respectively (stage I-case8, stage II-case1, case4 and case6, stage III-case5 and case7, stage IV-case2 and case3). **e** Kaplan−Meier analysis showing the correlations between ASPP2 expression and the overall survival of patients with GBC, determined by Log-Rank test. ASPP2 low, *n* = 50; ASPP2 high, *n* = 22. *P *<* 0.05, **P *<* 0.01, ***P *<* 0.001. Data are derived from three independent experiments and presented as means ± SDs

To explore the clinical significance of ASPP2 in human GBC, we first examined the expression of ASPP2 by immunohistochemistry (IHC), western blotting, and quantitative real-time polymerase chain reaction (qPCR) in GBC tissues and paired normal gallbladder tissues. IHC analysis showed that ASPP2 expression was significantly lower in GBC tissues than in 72 pair-matched normal tissues, both in the cytoplasm and nucleus (Fig. 1a). Downregulation of ASPP2 was further confirmed at the protein and mRNA levels in representative eight paired GBC tissues and normal tissues (Fig. 1c, d). In addition, we investigated whether the expression of ASPP2 was correlated with clinicopathological characteristics and prognosis in human GBC. Notably, reduced expression of ASPP2 was significantly associated with advanced TNM stage (*χ*^2^ = 33.513, *P* *<* 0.001), lymph node metastasis (*χ*^2^ = 19.415, *P* *<* 0.001), and poor tumor differentiation (*χ*^2^ = 25.222, *P* *<* 0.001) in GBC (Fig. 1b and Table 1). Kaplan−Meier analysis showed that patients with low ASPP2 expression exhibited a shorter overall survival (OS) than those with high ASPP2 expression (Fig. 1e). Cox’s multivariate analysis indicated that downregulation of ASPP2 was an independent prognostic risk factor for OS in patients with GBC (Supplementary Table S3). Moreover, gene set enrichment analysis was used to investigate the correlation between ASPP2 and several other tumors in the public database *The Cancer Genome Atlas* (TCGA), and the results showed that ASPP2 was involved in cell adhesion and tight junctions, which are related to tumor metastasis (Fig. S1). Taken together, these observations suggested that ASPP2 levels were frequently downregulated in human GBC and that ASPP2 may promote the EMT and metastasis.

**Table 1:** Correlation between ASPP2 and clinicopathologic characteristics in patients with GBC

Characteristic	No.	ASPP2		*P*
		Low	High	
Age				0.638
<60	39	28	11	
≥60	33	22	11	
Gender				0.949
Male	20	14	6	
Female	52	36	16	
Tumor/normal tissue				**<0.001**
Tumor	72	50	22	
Normal	72	12	60	
Lymph node metastasis				**<0.001**
Present	41	37	4	
Absent	31	13	18	
TNM stage				**<0.001**
I/II	24	6	18	
III/IV	48	44	4	
Differentiation				**<0.001**
Well	18	4	14	
Moderate/poor	54	46	8	

### ASPP2 deficiency promoted the proliferation, migration, and invasion of GBC cells

**Fig. 2:**
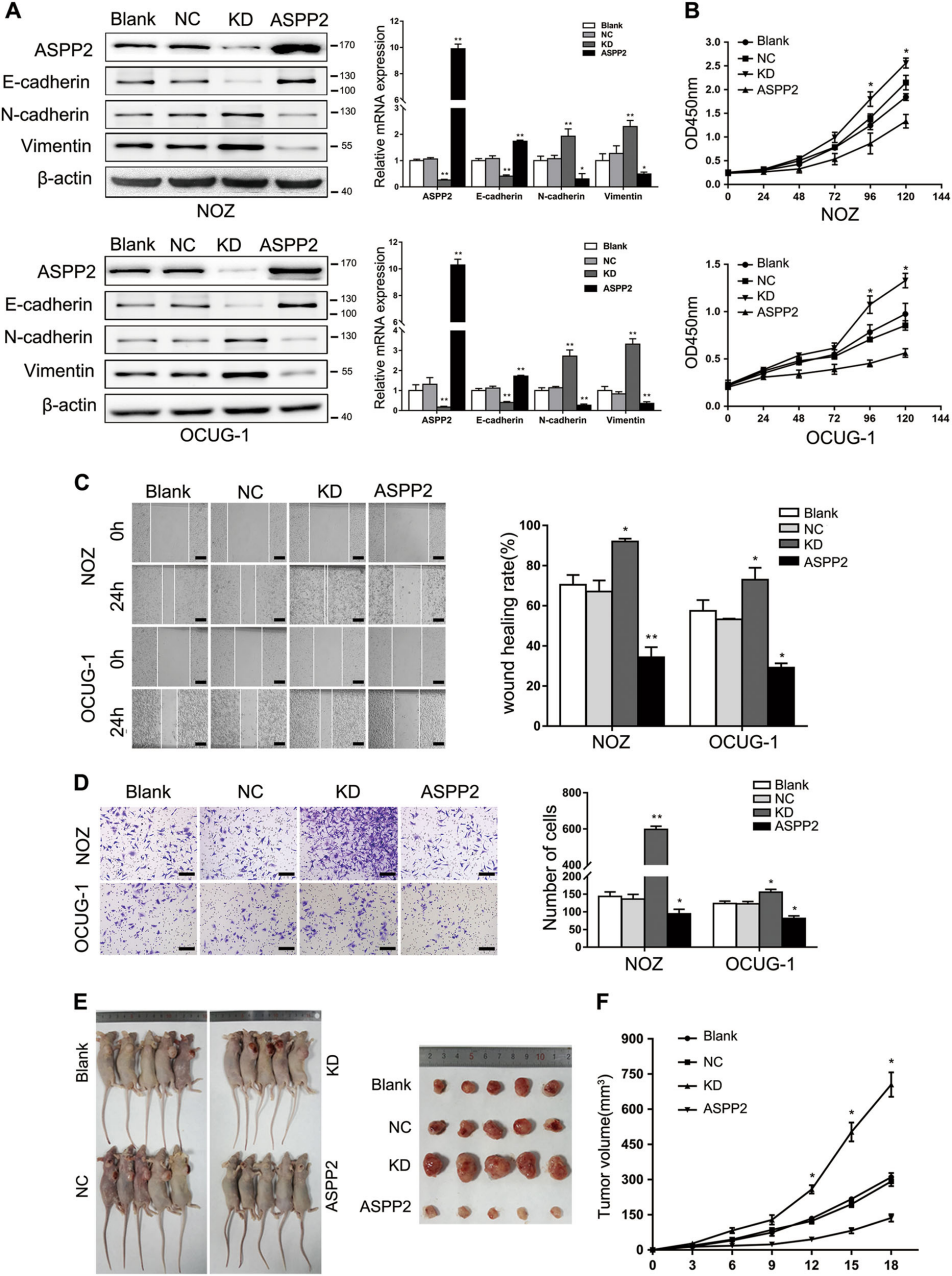
ASPP2 deficiency promoted the proliferation, migration, and invasion of GBC cells in vitro and in vivo. **a** Western blotting and qPCR were performed to detect the expression levels of ASPP2, E-cadherin, N-cadherin, and vimentin in NOZ and OCUG-1 cells with lentivirus-mediated ASPP2 knockdown (KD) or overexpression (ASPP2). A lentiviral vector containing an siRNA that did not recognize any human gene was constructed as a negative control (NC). Cells without any treatment were used as the blank control (Blank). **b** CCK-8 assays were conducted to analyze the proliferative ability of GBC cells. **c** Wound healing assays were used to investigate the migration ability of the cells. Representative images are shown in the left panel, and the results were compared statistically in the right panel. Scale bar, 500 μm. **d** Transwell assays were performed to measure the invasion ability of GBC cells. Scale bar, 500 μm. **e** Representative images of xenograft tumors from the indicated GBC cells (*n* = 5 mice per group). **f** Line chart showing the volume of xenograft tumors from the indicated GBC cells. *P *<* 0.05, **P *<* 0.01, determined by one-way ANOVA and independent Student’s *t* tests. Data are derived from three independent experiments and presented as means ± SDs. OD optical density

To elucidate whether ASPP2 affected the biological characteristics of GBC, we first evaluated ASPP2 expression in three GBC cell lines (NOZ, OCUG-1, and GBC-SD). We found that ASPP2 expression was decreased in all cell lines, especially in GBC-SD (Fig. S2A). Thus, NOZ and OCUG-1 cells were chosen for the subsequent studies. Then we stably established ectopic ASPP2 expression or knockdown (KD) GBC cell lines (NOZ and OCUG-1 cells; Fig. 2a). We found that ASPP2 KD induced EMT-like characteristics with respect to mRNA and protein expression profiles, including downregulation of E-cadherin and upregulation of N-cadherin and vimentin (Fig. 2a and Fig. S2B). However, ASPP2 KD or overexpression had minimal impact on the expression levels of other ASPP2-induced EMT markers, such as ZEB1 and β-catenin^13^, in GBC cells (Fig. S2C-D). Cell proliferation, migration, and invasion were also significantly increased after transfection with lentiviral shRNA targeting endogenous ASPP2. Interestingly, restoration of exogenous ASPP2 expression reversed the effects of ASPP2 KD, suggesting that ASPP2 may act as a switch for the EMT and control the mobility of GBC cells (Fig. 2b–d).

In vivo tumorigenicity assays showed that ASPP2 KD significantly increased the volumes of xenograft tumors compared with those in the blank and negative control groups. Moreover, these effects were reversed by expression of exogenous ASPP2 (Fig. 2e, f and Fig. S2E-F). Thus, our results indicated that ASPP2 deficiency could facilitate tumor cell proliferation, migration, and invasion in vitro and in vivo.

### ASPP2 KD promoted the recruitment of macrophages and facilitated tumor lung metastasis

**Fig. 3:**
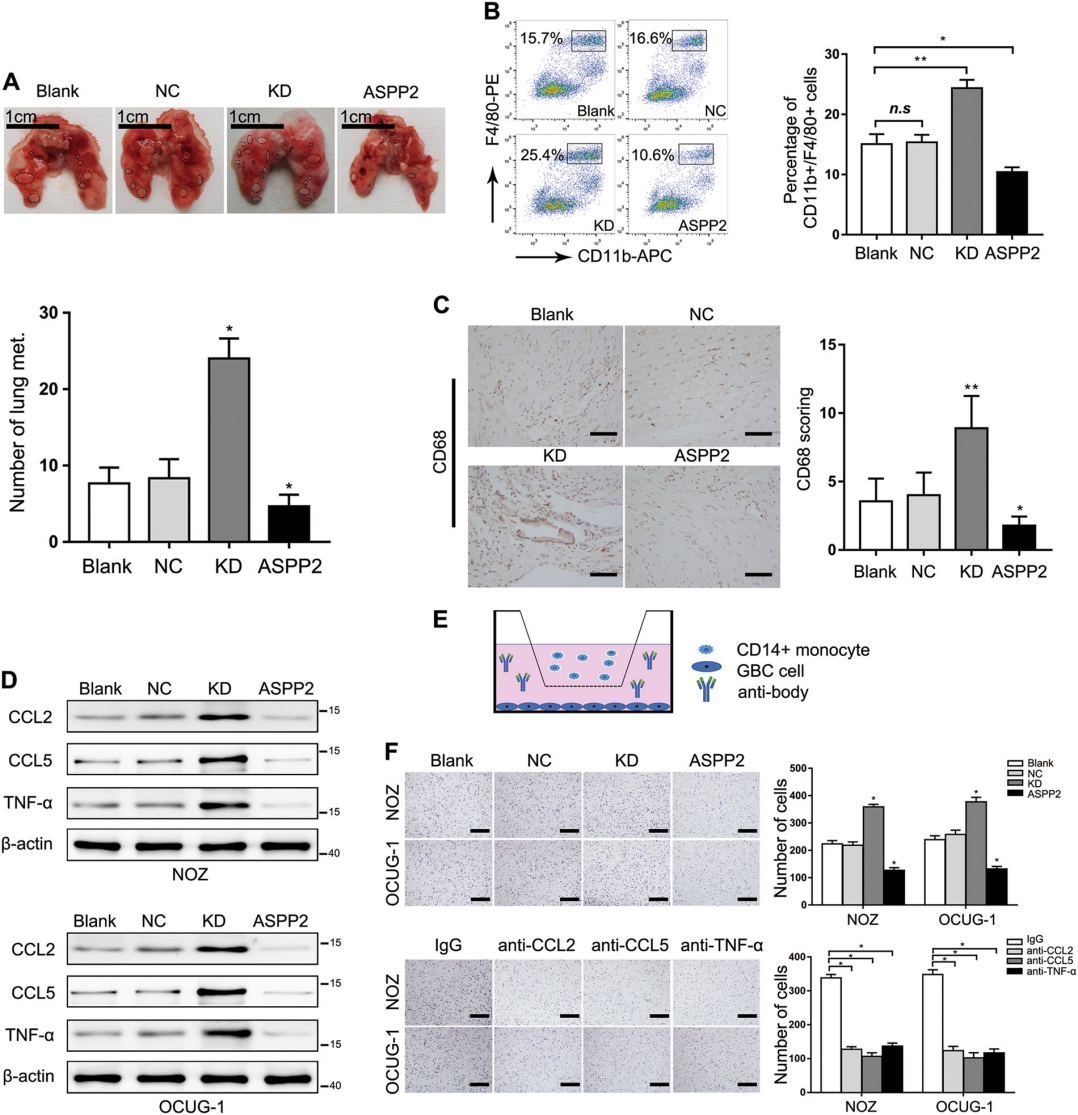
Knockdown of ASPP2 promoted macrophage recruitment and facilitated tumor lung metastasis. **a** Representative images of the lungs of mice treated with the indicated cells. The black dotted lines indicate lung metastases (*n* = 3 mice per group). **b** Flow cytometry analysis of CD11b^+^ and F4/80^+^ percentages in lung metastases derived from NOZ cells with the indicated treatment. **c** IHC staining showing CD68 expression in xenograft tumors after treatment with the indicated cells lines. *n* = 5. Scale bar, 50 μm. **d** Western blotting was performed to analyze the protein expression of CCL2, CCL5, and TNF-α in the indicated GBC cell lines. **e** Schematic diagram of the monocyte migration assay. **f** Transwell migration experiments in CD14^+^ monocytes attracted by the indicated GBC cells (upper) or with different neutralizing antibodies (bottom). Scale bar, 500 μm. *P *<* 0.05, **P *<* 0.01, determined by one-way ANOVA and independent Student’s *t* tests. Data are derived from three independent experiments and are presented as means ± SDs

Accumulating evidence has demonstrated that various stromal components, including macrophages, play important roles in tumor metastasis to the lung and in remodeling of the TME^6,25,26^. Consistent with this, we found that downregulation of ASPP2 significantly increased lung metastasis, whereas overexpression of ASPP2 inhibited lung metastasis (Fig. 3a). Next, we used flow cytometry analyses to examine CD11b^+^F4/80^+^ macrophages in lung metastases and found that ASPP2 KD enhanced, whereas ASPP2 overexpression suppressed macrophage recruitment (Fig. 3b), as confirmed by IHC in xenograft tumors (Fig. 3c and Fig. S3B). However, changes in macrophages were not due to alterations in their origin because the number of macrophages in the spleens and bone marrow of the mice remain unchanged (data not shown). Previous studies have reported that cytokines played an important role to remodel the TME and promote EMT. The cytokines C−C motif chemokine ligand (CCL) 2 and CCL5, which promote the recruitment of tumor-associated macrophages (TAMs)^27,28^, as well as interleukin (IL)-8, which is a ligand for C−X−C motif chemokine receptor (CXCR) 1 and CXCR2, are highly expressed on the surface of M2 macrophages, and TAMs have an M2-like phenotype^29^. However, ASPP2-induced changes in cytokines have not yet been extensively investigated. To determine the major cytokines regulated by ASPP2, we examined a series of cytokines and explored whether ASPP2 could induce cytokine secretion to modulate the TME (Fig. S3A). Interestingly, we found that ASPP2 KD, but not overexpression, increased the expression of CCL2, CCL5, and tumor necrosis factor (TNF)-α and enhanced their secretion in GBC cells (Fig. 3d; Fig. S3C-E). Furthermore, cell migration assay revealed that ASPP2 KD enhanced, while ASPP2 overexpression suppressed, the chemoattraction of GBC cells to CD14^+^ monocytes. Addition of neutralizing antibodies attenuated monocyte migration (Fig. 3e, f). Therefore, the above results showed that reducing ASPP2 expression promoted the recruitment of macrophages and facilitated tumor lung metastasis.

### TAM recruitment was negatively correlated with ASPP2 expression and induced EMT-like changes

**Fig. 4:**
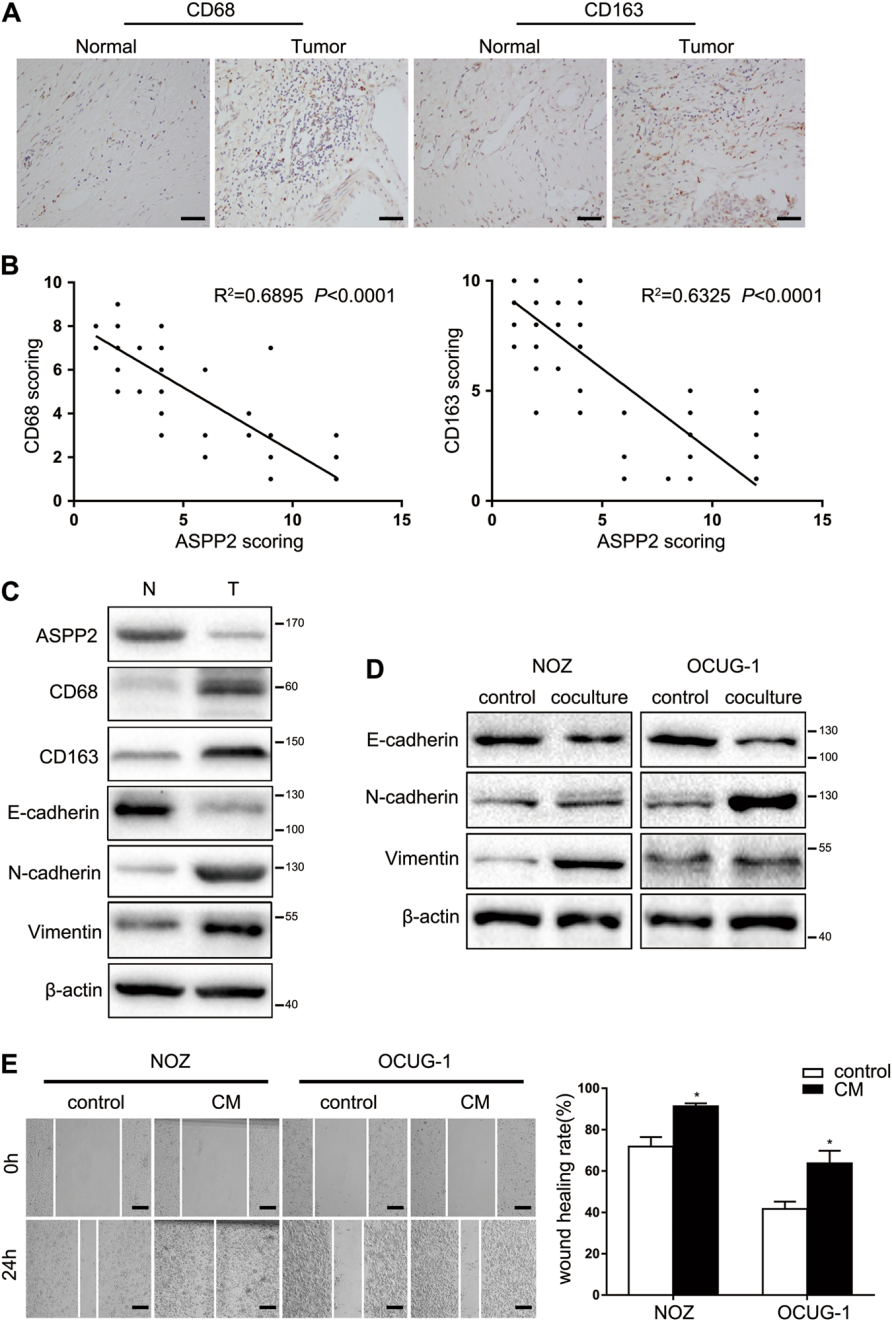
TAM recruitment was negatively correlated with ASPP2 and induced EMT-like changes. **a** Representative images of IHC staining for CD68 and CD163 expression in 72 human GBC samples and adjacent nontumor tissues. Scale bar, 50 μm. **b** Linear regression was used to analyze the correlations between ASPP2 and the TAM markers CD68 and CD163. **c** Western blotting was performed to detect the expression of ASPP2, CD68, CD163, E-cadherin, N-cadherin, and vimentin in representative GBC tissues (T) and pair-matched normal tissues (N). **d** Expression of E-cadherin, N-cadherin, and vimentin in GBC cells cultured with normal medium or cocultured with macrophages. **e** Wound healing assays were performed to measure the invasion ability of GBC cells with normal medium or conditioned medium (CM). Scale bar, 500 μm. *P *<* 0.05. Data are derived from three independent experiments

Next, we further explored the correlations between TAMs and clinical features in GBC specimens and cells. The expression levels of CD68 and CD163, representative cell markers of TAMs, were significantly higher in GBC tissues than in pair-matched normal tissues (Fig. 4a). Next, we investigated whether ASPP2 expression was correlated with TAM recruitment in cancer tissues. The results showed that low ASPP2 expression was associated with increased infiltration of TAMs (Fig. 4b). In addition, we also found EMT-like changes in GBC samples in which CD68 and CD163 were highly expressed (Fig. 4c). E-cadherin expression was reduced, whereas N-cadherin and vimentin levels were increased in GBC cells following coculture with macrophages (Fig. 4d). Cell migration was also increased after coculture with the conditioned medium from macrophages (Fig. 4e). Therefore, these observations demonstrated that downregulation of ASPP2 was associated with TAM recruitment and poor prognosis in patients with GBC. TAM recruitment was negatively correlated with ASPP2 and induced EMT-like changes.

### ASPP2 regulated the expression of aPKC-ι and GLI1

**Fig. 5:**
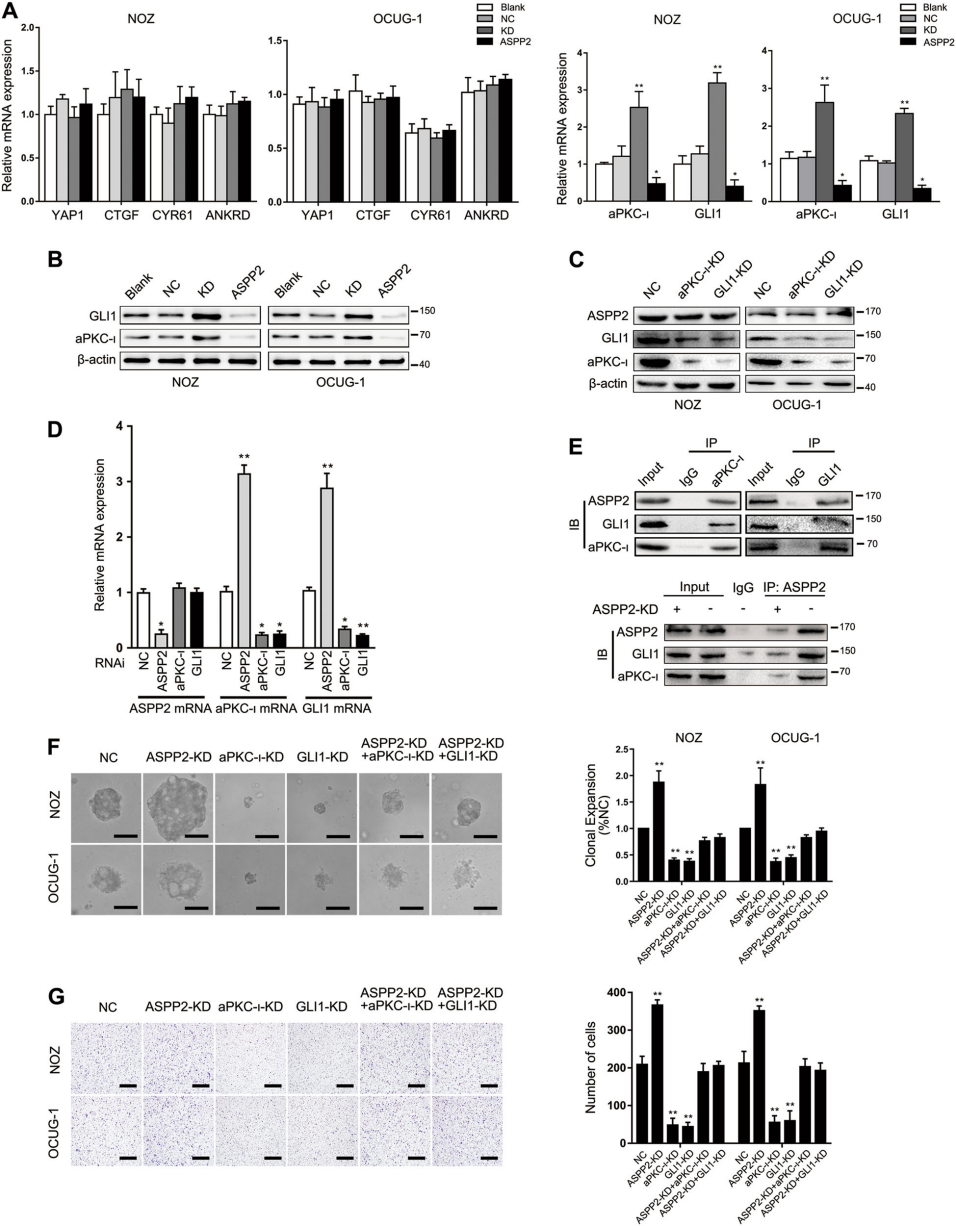
ASPP2 regulated the expression of aPKC-ι and GLI1. **a** qPCR analysis for expression of YAP1, CTGF, CYR61, ANKRD, aPKC-ι, and GLI1 in the indicated GBC cells. **b** Western blotting was used to analyze the protein expression of aPKC-ι and GLI1 in the indicated GBC cells. **c** Western blotting was used to analyze the protein expression of ASPP2, aPKC-ι, and GLI1 in aPKC-ι KD or GLI1 KD cells. **d** qPCR was used to analyze the expression of ASPP2, aPKC-ι, and GLI1 with RNAi treatment in GBC cells. **e** Co-IP assays were performed to detect the interaction among ASPP2, aPKC-ι, and GLI1 in GBC cells. **f** Anchorage-independent growth was evaluated by soft agar growth assays in the indicated GBC cells. Scale bar, 50 μm. **g** Transwell migration experiments in CD14^+^ monocytes attracted by the indicated GBC cells. Scale bar, 500 μm. *P *<* 0.05, **P *<* 0.01, determined by independent Student’s *t* tests. Data are derived from three independent experiments and presented as means ± SDs

Previously, we reported that aPKC-ι is a critical polarization regulator and induces the EMT in CCA^30^. Interestingly, aPKC-ι also regulates YAP1 nuclear localization and activates the Hh signaling pathway to promote tumor growth^31,32^. These findings suggest that aPKC-ι may interact with multiple signals involved in tumor progression. Here, we hypothesized that aPKC-ι may participate in regulation of the EMT via ASPP2. Surprisingly, we found that ASPP2 KD increased the expression of aPKC-ι at both the mRNA and protein levels. In addition, ASPP2 deficiency significantly increased the expression of GLI1, a major Hh transcriptional regulator, but did not affect YAP1 and its target genes *CTGF*, *CYR61*, and *ANKRD* in GBC cell lines. Consistent with this, the expression levels of aPKC-ι and GLI1 were restored by exogenous ASPP2, which was confirmed by qPCR and immunoblotting (Fig. 5a, b).

Based on these findings, we then investigated whether aPKC-ι or GLI1 affected the expression of ASPP2. When endogenous aPKC-ι or GLI1 was knocked down, GLI1 or aPKC-ι expression, but not ASPP2 expression, was significantly decreased (Fig. 5c). ASPP2, aPKC-ι, and GLI1 RNAi led to efficient knockdown of their respective target. ASPP2 RNAi significantly increased aPKC-ι and GLI1 expression. aPKC-ι and GLI1 RNAi also exhibited reduced GLI1 and aPKC-ι expression, but did not affect ASPP2 expression (Fig. 5d). Next, immunoprecipitation experiments were performed to investigate whether ASPP2 affected binding with aPKC-ι and GLI1. The results showed that aPKC-ι and GLI1 immunoprecipitated with ASPP2. The reciprocal immunoprecipitation confirmed this interaction. More importantly, ASPP2 KD attenuated the ASPP2−aPKC-ι−GLI1 interaction (Fig. 5e). Furthermore, downregulation of ASPP2 increased, while knockdown aPKC-ι (aPKC-ι-KD) or GLI1 (GLI1-KD) reduced anchorage-independent growth by soft agar growth assays in GBC cells. Co-downregulation of ASPP2 with aPKC-ι or GLI1 reversed this effect (Fig. 5f). Consistently, we found that aPKC-ι-KD or GLI1-KD suppressed the chemoattraction of GBC cells to CD14+ monocytes, which was abolished by ASPP2 KD (Fig. 5g). Taken together, these findings suggested that ASPP2 may indirectly activate GLI1 expression, in part through aPKC-ι, thereby regulating the EMT and TME.

### Depletion of ASPP2 regulated GLI1 transcriptional activity via aPKC-ι

**Fig. 6:**
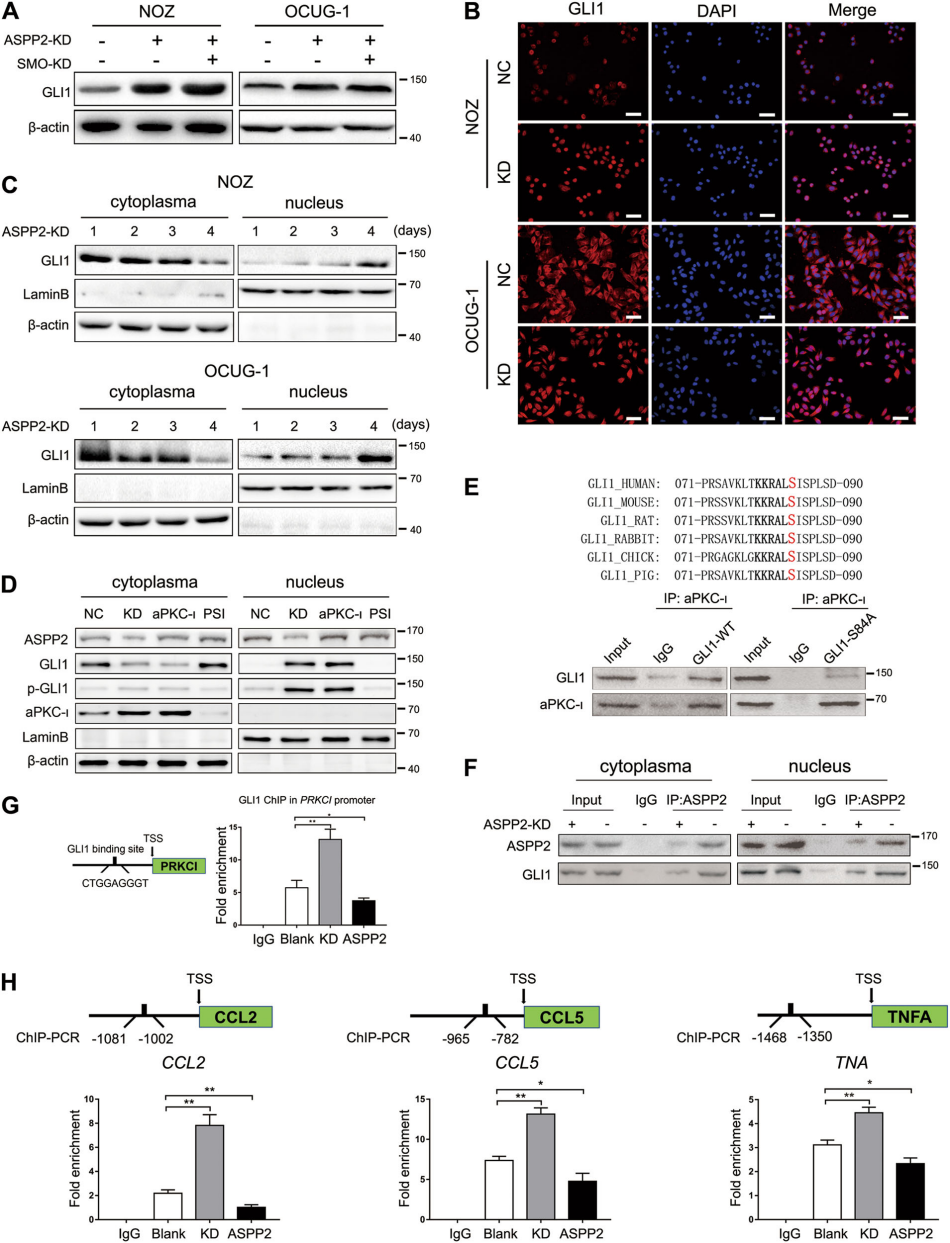
Depletion of ASPP2 regulated GLI1 transcriptional activity via aPKC-ι. **a** Western blotting was used to detect the expression of GLI1 in ASPP2 knockdown (KD) or SMO knockdown GBC cells. **b** Immunofluorescence analysis demonstrating the shift in GLI1 expression between the nucleus and cytoplasm in GBC cells with the indicated treatments. Scale bar, 50 μm. **c** Western blot analysis of nuclear and cytoplasmic extracts of GBC cells after treatment with lentivirus vectors for the indicated times. **d** The distributions of ASPP2, GLI1, phospho-GLI1, and aPKC-ι were detected by analysis of nuclear and cytoplasmic fractions in GBC cells. **e** Upper panel, the conserved structure in GLI1. Lower panel, Co-IP assays were performed to detect the interaction between aPKC-ι and GLI1 in GLI1-WT or GLI1-S84A cells. **f** Co-IP assays were performed to detect the interaction between ASPP2 and GLI1 in the cytoplasm and nucleus of NOZ cells with the indicated treatment. **g** Left panel, GLI1 binding site within the promoter region of aPKC-ι. Right panel, GLI1 ChIP in aPKC-ι promoter. **h** ChIP assays were used to analyze the enrichment of GLI1 on the promoters of *CCL2*, *CCL5*, and *TNF-α* in GBC cells transfected with ASPP2-knockdown (KD) or ASPP2-overexpression (ASPP2). *P *<* 0.05, **P *<* 0.01, determined by independent Student’s *t* tests. Data are from three independent experiments and presented as means ± SDs

Although previous studies have reported that Hh signaling may be a potential therapeutic target for GBC, published preclinical data are limited^33^. Cyclopamine, an inhibitor of SMO, has been shown to exert few effects in several cancers, and the mechanism mediating aberrant activation of the Hh pathway in GBC remains unknown. In fact, we found that ASPP2 deficiency or overexpression had little effect on the expression of PTCH1 and SMO (Fig. S4A-B). Thus, we next examined whether GBCs were characterized by noncanonical, SMO-independent, GLI1 activation. Interestingly, in GBC cells, cyclopamine did not inhibit ASPP2 KD-induced GLI1 activity (Fig. S4C). Moreover, si-SMO had limited effects on ASPP2 KD-induced GLI1 activity (Fig. 6a and Fig. S4D). In GBC cells, we found that ASPP2 KD induced GLI1 nuclear localization, which increased with time after lentiviral transfection (Fig. 6b, c and Fig. S4E). Consistent with this, IHC results showed that ASPP2 induced nuclear accumulation of GLI1 in xenograft tumors (Fig. S4F).

Because aPKC-ι is a serine/threonine kinase that regulates GLI activity in basal cell carcinoma^34^, we asked whether aPKC-ι mediated nuclear localization of GLI1 through phosphorylation. Indeed, we found that phosphorylation of GLI1 had a significant impact on the distribution of GLI1 in the cytoplasm and nucleus as endogenous or exogenous levels of aPKC-ι increased. In addition, the phosphorylation of GLI1 was largely inhibited by treatment with PSI, an aPKC peptide inhibitor. Of note, in case of ASPP2 knockdown or aPKC-ι overexpression in NOZ cells, we did not detect aPKC-ι in the nucleus (Fig. 6d). Previous studies reported that 79-KKRALS-84 is highly conserved in GLI1, as a potential phosphorylation site^24^. Thus, we hypothesized that Ser84 may be phosphorylated by aPKC-ι. Then, we generated wild-type GLI1 (GLI1-WT) and Ser84 nonphosphorylatable mutant GLI1 (GLI1-S84A). Immunoprecipitation experiments showed that GLI1-WT bound significantly more aPKC-ι than GLI1-S84A (Fig. 6e). As shown in Fig. 1a and Fig S2B, we found that ASPP2 was expressed in both cytoplasm and nucleus of GBC cells. Intriguingly, recent studies have suggested that the subcellular localization of ASPP2 may have specific functions^35^. This prompted us to test if ASPP2 regulated GLI1 in the nucleus. The results showed that ASPP2 deficiency attenuated the ASPP2−GLI1 interaction in the nucleus of NOZ cells (Fig. 6f). Interestingly, consistent with previously studies, we also found that GLI1 directly bound to the promoter regions of *PRKCI* (aPKC-ι is encoded by the *PRKCI* gene) (Fig. 6g). Moreover, chromatin immunoprecipitation (ChIP) assays confirmed the direct binding of GLI1 to the promoters of *CCL2*, *CCL5*, and *TNFA* (Fig. 6h). Knockdown ASPP2 may enhance recruitment of TAMs by modulating transcriptional activity of these target genes. Taken together, these results suggested that ASPP2 regulated GLI1 nuclear translocation through the noncanonical Hh pathway involving aPKC-ι-mediated GLI1 phosphorylation. ASPP2 deficiency decreased the ASPP2−GLI1 interaction and facilitated the binding of GLI1 to the promoter of downstream target genes. aPKC-ι and GLI1 may form a positive feedback loop to amplify the signal.

### aPKC-ι and GLI1 were associated with poor prognosis in patients with GBC and modulated the secretion of chemokines

**Fig. 7:**
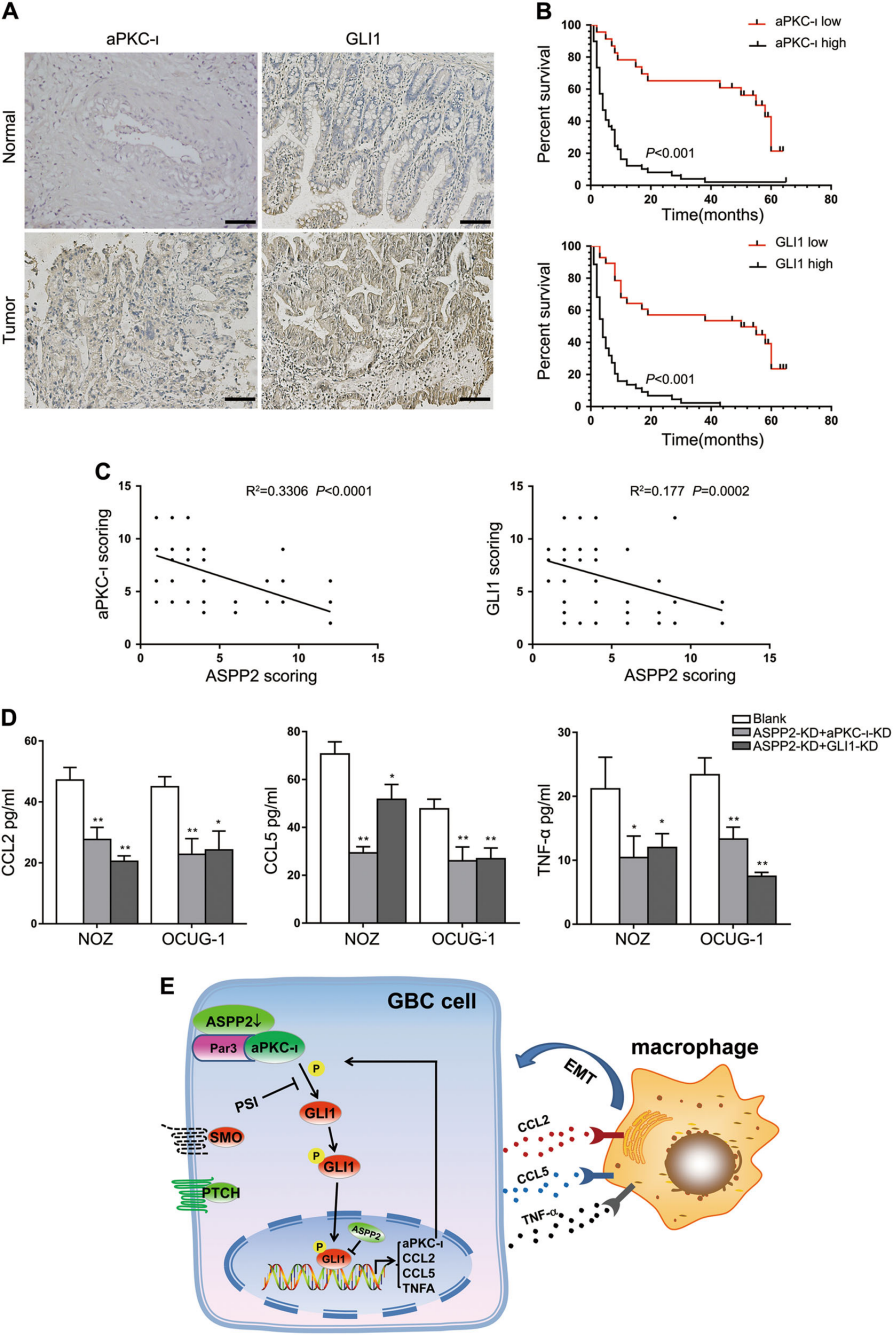
aPKC-ι and GLI1 were associated with poor prognosis in patients with GBC and modulated the secretion of chemokines. **a** IHC staining showing aPKC-ι or GLI1 expression in 72 human GBC samples and pair-matched normal tissues. Scale bar, 50 μm. **b** Kaplan−Meier analysis indicating the correlation between aPKC-ι or GLI1 expression and overall survival in patients with GBC. **c** Linear regression was used to analyze the correlations between ASPP2 and aPKC-ι or GLI1. **d** ELISA was used to analyze the secretion of CCL2, CCL5, and TNF-α into the supernatants of GBC cell cultures after transfection with aPKC-ι-siRNA or GLI1-siRNA. **e** Schematic summarizing how ASPP2 regulated invasion and metastasis via the aPKC-ι-GLI1 pathway and enhanced macrophage recruitment in GBC. *P *<* 0.05, **P *<* 0.01, determined by independent Student’s *t* tests. Data are derived from three independent experiments and are presented as means ± SDs

Finally, we further confirmed the relationships among ASPP2, aPKC-ι, and GLI1 in GBC samples. The results showed that aPKC-ι and GLI1 expression levels were significantly higher in GBC tissues than in pair-matched normal tissues and were negatively correlated with ASPP2 expression (Fig. 7a–c). The expression levels of aPKC-ι and GLI1 were significantly associated with advanced TNM stage (*χ*^2^ = 19.965, 15.458, respectively; *P* *<* 0.001), lymph node metastasis (*χ*^2^ = 13.125, 15.044, respectively; *P* *<* 0.001), and poor tumor differentiation (*χ*^2^ = 29.154, 15.273, respectively; *P* *<* 0.001) in GBC (Supplementary Table S2). Multivariate Cox regression analyses indicated that aPKC-ι and GLI1 were independent prognostic factors for OS in patients with GBC (Supplementary Table S3). Furthermore, we found that the expression of ASPP2 in some tumor tissues was almost equivalent to the normal tissues (Fig. 1c). Then, we further compared ASPP2 expression with GLI1 expression and macrophage infiltration in representative samples. The IHC analysis confirmed that low expression of ASPP2 correlated with increased GLI1 expression and TAMs infiltration in GBC tissues, while high expression of ASPP2 exhibited opposite results (Fig. S5A-B).

Next, we investigated the effects of aPKC-ι and GLI1 on changes in chemokine levels in the supernatants of GBC cell cultures. Our data suggested that downregulation of aPKC-ι or GLI1 reduced the secretion of CCL2, CCL5, and TNF-α, which further supported our previous results (Fig. 7d). Taken together, our results strongly suggested that ASPP2 affected GBC invasion and metastasis through the aPKC-ι/GLI1 signaling pathway and remodeling of the TME.

## Discussion

Tumors develop in a complex environment, which they depend on for invasion and metastasis. The TME is also thought to play an important role in crosstalk with cancer cells^36,37^. Although previous studies have shown that ASPP2 is correlated with prognosis in multiple types of cancer, the relationships and molecular mechanisms through which ASPP2 interacts with the TME remain unknown.

Here, we found that downregulation of ASPP2 promoted GBC invasion and metastasis through the aPKC-ι/GLI1 pathway and enhanced macrophage recruitment. Previous studies have focused on the transactivity effects of p53 through ASPP2 in the nucleus, while accumulating evidence has highlighted the tumor-suppressive role of ASPP2 crosstalk with other pathways^35,38^. In this study, our data demonstrated that ASPP2 was frequently downregulated in GBC tissues compared with that in normal tissues. Moreover, multivariate Cox regression analyses suggested ASPP2 was an independent prognostic factor for the OS of patients with GBC. These clinical data strongly indicated that ASPP2 could be an important prognostic biomarker for patients with GBC.

In this study, we found that ASPP2 may act as a molecular switch for the EMT/MET and control tumor progression. Our findings are supported by other studies demonstrating that ASPP2 can regulate epithelial plasticity and cell−cell adhesion in vitro and in vivo through different signal pathways^13^. However, tumor progression is a complex process, and bidirectional communication between cancer cells and the TME is critical for tumor progression and metastasis^25^. In these contexts, we hypothesized that ASPP2 may also affect the microenvironment via a special mechanism. As important regulators of tumorigenesis, macrophages are major components of the TME. Numerous studies have demonstrated that tumor cells can recruit monocytes to infiltrate the tumor tissue by secreting chemokines and differentiate into TAMs. Interestingly, ASPP2 KD, but not overexpression, increased the expression of *CCL2*, *CCL5*, and *TNF-α* and enhanced their secretion, which may promote macrophage recruitment to tumor tissues and support multiple aspects of tumor progression. Our work is consistent with several previous studies demonstrating that these chemokines can promote the recruitment of TAMs to remodel the TME by the “yin and yang” effect or a positive feedback loop between cancer cells and macrophages^29,39^.

However, care should be taken when interpreting our findings that macrophages can be induced to TAMs. Although our data showed that ASPP2 KD induced an M2-like phenotype in macrophages to promote lung metastasis of cancer cells, which was also supported by previous studies, the roles of these cells in metastatic progression cannot be ruled out. Moreover, it is difficult to simply classify TAMs into M1 or M2 states in the complex TME in vivo, although the cells can be induced into M1 or M2 macrophages in vitro. Indeed, large-scale transcriptome analysis supported that TAMs have a mixed phenotype of both M1 and M2 macrophages in vivo^39,40^. In addition, IL-6 and IL-10, which act as cytokines for M2-like macrophages, were not upregulated following ASPP2 KD in GBC cells. Further studies are needed to determine whether these cytokines induce M2-like macrophages through activation of other immune cells in the TME, such as Th2 cells, or through a noncanonical pathway.

In the present study, we found that ASPP2 regulated the expression of aPKC-ι, which has been shown to affect the nuclear localization of the transcription factor YAP1 in ovarian cancer. Thus, we assessed whether ASPP2 and aPKC-ι activated a similar signaling pathway in GBC cells. Surprisingly, our data suggested that ASPP2 and aPKC-ι did not affect the expression of YAP1 and target genes, but regulated GLI1, the major mediator of Hh signaling. Interestingly, our results suggested that GLI1 expression in GBC cells may be driven by an ASPP2/aPKC-ι pathway rather than canonical Hh signaling. It is also noteworthy that ASPP2 was expressed in both cytoplasm and nucleus of GBC cells. In the cytoplasm, ASPP2 KD promoted GLI1 nuclear translocation via aPKC-ι-mediated GLI1 phosphorylation. In the nucleus, ASPP2 depletion facilitated the dissociation of GLI1 from ASPP2, which led to activation of GLI1 target genes. aPKC-ι, as a target of GLI1, also activated Hh signaling through a positive feedback loop. However, the nuclear import mechanism of ASPP2 remains enigmatic. In addition, further studies are needed to elucidate the mechanisms mediating the interaction between cytokines secreted by tumor cells and macrophages.

In conclusion, our study highlighted the importance of ASPP2 downregulation in promoting the invasion and metastasis of GBC cells via a noncanonical Hh pathway, i.e., the aPKC-ι/GLI1 signaling pathway, activating downstream cytokines to recruit macrophages that could then infiltrate into the tumor tissues. Accordingly, our findings expand our knowledge of the functions of ASPP2 in cancer, including its role as a regulator of the EMT in GBC and as an important upstream factor in the TME. These results provide important insights into the development of new, effective therapeutic approaches for the treatment of GBC.

## Materials and methods

### Patients and specimens

In total, 72 human GBC tissues and paired normal gallbladder tissues (5 cm distant from tumor) were collected from patients undergoing resection at the Department of Biliary and Pancreatic Surgery, Tongji Hospital (Wuhan, China) between January 2009 and December 2016. Ethical approval for the use of human samples was obtained from the Tongji Hospital Research Ethical Committee. None of the patients had received any adjuvant therapy before surgery. All cases were diagnosed by two independent pathologists. The detailed clinicopathological characteristics of the 72 patients with GBC are listed in Supplementary Table S1.

### Immunohistochemistry

The expressions of ASPP2, aPKC-ι, GLI1, CD68, and CD163 were detected by immunostaining as previously reported^41^. The positively stained cells were determined in at least five areas at ×400 magnification and assigned to one of the following categories: score 0 (negative); score 1 (<25% positive cells in a specimen); score 2 (25–50% positive cells in a specimen); score 3 (50–75% positive cells in a specimen); score 4 (>75% positive cells in a specimen). The intensity of immunostaining was scored as follows: 1+ (weak); 2+ (moderate), and 3+ (intense). The above two values are multiplied to produce a total score. The total score ≤4 was considered as low expression and >4 as high expression^42^. Evaluation of immunostaining was performed by three independent experienced investigators who were blinded to the patient conditions.

### Cell lines

Human GBC cell lines NOZ, GBC-SD, and OCUG-1 were generously provided by Prof. Yingbin Liu, Xinhua Hospital, Shanghai Jiao Tong University School of Medicine, China. These cells were maintained in William’s medium, RPMI 1640 or Dulbecco’s modified Eagle medium (both from Gibco, Grand Island, NY, USA) supplemented with 10% fetal bovine serum (Gibco). All cell lines were authenticated, mycoplasma-free and cultured at 37 °C in a humidified incubator containing 5% CO_2_.

### Animal study

Six-week-old female BALB/c-nude mice were divided into four groups (*n* = 5 mice per group) and housed under specific pathogen-free conditions in Central Animal Laboratory, Tongji Medical College. The mice were injected subcutaneously in the upper back with 5×10^6^ transfected GBC cells. The diameter of tumors and the weight of the mice were measured every 3 days. The volumes of the tumor were calculated using the formula: 1/2 (length × width^2^). For lung metastasis model, the GBC cells (2×10^6^) were suspended in serum-free medium and injected into tail vein of mice (*n* = 3 mice per group). All mice were sacrificed 3 weeks later. The number of lung metastases was counted under a microscope. Animal welfare and experimental procedures were carried out in accordance with the ARRIVE (Animal Research: Reporting In Vivo Experiments) guidelines and were approved by the Committee on the Ethics of Animal Experiments of the Tongji Medical College, HUST.

Additional experimental procedures are provided in detail in the Supplementary data.

### Statistical analyses

Statistical analyses were performed using SPSS 22.0 software (IBM SPSS, Armonk, NY, USA) or GraphPad Prism 6.0 (GraphPad Software, La Jolla, CA, USA). The results were presented as the mean ± standard deviation (SD). Quantitative data were analyzed by two-tailed independent Student’s *t* tests and analysis of variance. Categorical variables were compared using chi-square tests or Fisher exact tests. Clinical correlations were analyzed using *χ*^2^ tests, and survival analysis was conducted by the Kaplan−Meier method with log-rank tests. The univariate and multivariate analyses were assessed using a Cox proportional hazards model. Differences with *P* values of less than 0.05 were considered statistically significant.

## Electronic supplementary material

Supplementary Experimental Procedures

Supplemental Figure Legends

Figure S1

Figure S2

Figure S3

Figure S4

Figure S5

Supplementary tables

The STR authentication of GBC cell lines
