# Supplementary material for: Downregulation of ASPP2 promotes gallbladder cancer metastasis and macrophage recruitment via aPKC-ι/GLI1 pathway
Source: Cell Death Dis. 2018 Nov 2;9(11):1115. doi: 10.1038/s41419-018-1145-1 (PMC6214900; doi:10.1038/s41419-018-1145-1)
Supplement: Supplementary file 1 — Supplementary Experimental Procedures [file 41419_2018_1145_MOESM1_ESM.docx]

**Supplementary Experimental Procedures**

**1. *Lentiviral vector construction and transfected***

Lentiviral vector containing human ASPP2-cDNA, ASPP2-shRNA, which had antibiotic resistance to puromycin, were designed and purchased from Genechem Company Ltd (Shanghai China). The target sequences are described in the Supplementary Table S5. A lentiviral vector containing an shRNA that did not recognize any human gene was constructed as a negative control (NC). Lentiviral transfection was conducted according to the GenePharma Recombinant Lentivirus Operation Manual (http://www.genepharma.com). GBC cells were selected for 2 weeks in the presence of puromycin (2μg/ml) after transfected. The expression level of ASPP2 in GBC cell lines were confirmed by qPCR and western blotting.

**2. *Western blotting***

Western blotting assay was performed to detect the expression of ASPP2, aPKC-ι, GLI1 and EMT markers as previously described. Cytoplasmic and nuclear fractions were isolated by Nuclear and Cytoplasmic Protein Extraction Kit (Beyotime, Hangzhou, China) according with the manufacturer’s protocol. β-actin was used as a loading control for cytoplasmic fraction, whereas LaminB was used as a loading control for the nuclear fraction. The intensity of bands was evaluated by ImageJ2 (National Institute of Mental Health, Bethesda, MD, USA). The antibodies used in this study are listed in the Supplementary Table S4.

**3.*Quantitative real-time PCR***

The total RNA was extracted from GBC specimens or cell lines by using Trizol reagent (Invitrogen, USA) and the complementary DNA (cDNA) was synthesized with PrimeScript™ RT Master Mix (Takara Bio Inc, Dalian, China) following the manufacturer’s instructions. Quantitative real-time PCR (qPCR) was performed using SYBR® Premix EX Taq kit (Takara Bio Inc, Dalian, China) according to the standard protocol. The results were analyzed by using the comparative Ct method as previously described. The primers used in this study are listed in the Supplementary Table S5.

**4. *Cell proliferation assay***

A Cell Counting Kit-8 (Dojindo Laboratories Co. Ltd, Kumamoto, Japan) assay was used to measure the proliferation of cells with a plate reader (Bio-Tek Elx 800, USA). In brief, NOZ or OCUG-1cells were seeded in 96-well plates at a density of 5×10^3^ cells/well with complete culture medium at 37°C. Each group had triplicates (n=3). The proliferation of cell was detected at 24h, 48h, 72h, 96h and 120h. At the predetermined time, 10 μl CCK-8 solution was added into each well. After incubating for 1-2 hours at 37°C, absorbance was measured using the plate reader at 450nm according to the standard protocol.

**5. *Wound healing assay***

For wound healing assay, GBC cells were seeded in 6-well plates at a density of 5×10^5^ cells/well with complete culture medium at 37°C for 2-3 days. The confluent monolayer cells were scratched with a sterile 200-µL pipette tip, and the extent of closing wound gap was measured at 0h and 24h. Triplicate wells were used for each group.

**6. *Transwell assays***

For Transwell assay, Transwell chambers (8μm, Corning, NY, USA) were used. 1×10^4^ cells were seeded into the upper chamber containing 200 μl low serum (0.1% FBS) culture medium. The bottom chamber were filled with 600 μl complete culture medium (10% FBS). After incubating for 24h, the upper chamber were fixed with 4% paraformaldehyde for 30 min at room temperature; then, the cells on the upper side of the membrane were stained and counted using Image-Pro Plus v6.0 software package (Media Cybernetics Inc., MD, USA). Three independent samples were used per group.

**7. *Soft agar growth assay***

Soft agar growth assay was performed as described previously. Mixed 1.2% soft agar and 20% complete medium in equal volumes, and spread evenly on the bottom of 6-well plates. Cells were suspended in 0.7% soft agar with 20% complete medium and culture for 2 weeks. Photos were taken by using an Olympus BX51 microscope. All experiments were independently repeated three times.

**8. *Flow cytometry***

Flow cytometry was used to explore the effects of tumor cells on the recruitment of macrophages by analyzing specific markers CD11b^+^ or F4/80^+^. Anti-CD11b^+^ conjugated with APC (Biolegend, CA, USA), and anti-F4/80^+^ conjugated with PE (Biolegend, CA, USA), were used for flow cytometric analysis. All experiments were independently repeated three times.

**9. *Immunoprecipitation***

Co-immunoprecipitation (Co-IP) assay was carried out in GBC cells with different expression levels of ASPP2 as described previously. Briefly, the cells were lysed by RIPA buffer and divided into parallel groups named input or IPs. Then, the primary antibody or IgG was added to the lysates for incubating overnight at 4℃. Subsequently, the Protein A + G agarose beads were added to the mixture at at 4℃ for 3h. After centrifugation at 15,000 × g for 15 min, the beads were collected and washed 5 times with RIPA buffer. The immunoblotting was performed with the indicated antibodies as previously reported.

**10. *In vitro monocytes migration assay***

The human peripheral blood CD14^+^ monocytes were isolated from the healthy donors by high-gradient magnetic sorting using anti-CD14 microbeads (Miltenyi Biotec, Shanghai, China). Then, the migration of CD14^+^ monocytes was measured with a 5μm Transwell chamber. Briefly, CD14^+^ cells were plated into the upper chamber, and GBC cells were cultured in the lower chamber. After 24h, the number of cells on the lower side of the membrane was counted per field of view. All experiments were independently repeated three times.

**11. *Chromatin immunoprecipitation***

The Chromatin immunoprecipitation (ChIP) assay was performed as described. NOZ and OCUG-1 cells (1 × 10^7^) were cross-linked with 1% formaldehyde for 10 min and terminated by 125 mM glycine at room temperature. After centrifugation at 135 × g for 10 min, cells were washed with pro-cooling PBS for twice and added . protease inhibitors. Then, cells were lysed with lysis buffer, interrupted by sonicator, and then incubated with anti-GLI1 antibody or an IgG control at 4°C for 16h with rotation. Subsequently, incubation with 30µl of protein A+G agarose beads at 4°C for additional 2h, beads were washed in sequence with low salt buffer (0.1%SDS; 1% Triton X-100; 2mM EDTA; 20mM Tris-HCl，pH8.1; 150mM NaCl), high salt buffer (0.1%SDS; 1% Triton X-100; 2mM EDTA; 20mM Tris-HCl，pH8.1; 500mM NaCl), LiCl (1%NP40; 1% deoxycholate; 1mM EDTA; 10mM Tris-HCl，pH8.1), and another twice with TE (10mM Tris-HCl， 1mM EDTA pH8.0). Then, proteins binding to beads were eluted by 500µl elution buffer at 62°C for 2h with rotation. After protein digestion by 0.5 mg/ml proteinase K at 50°C for 2h, DNA was extracted by phenol/chloroform and precipitated by absolute alcohol. The purified DNA was subjected to qPCR using corresponding designed primers.

**12. Enzyme-linked immunosorbent assay**

Enzyme-linked immunosorbent assay (ELSIA) was performed as described previously. In brief, diluted CCL2, CCL5 or TNF--α was added into each well an 4℃ for overnight. Then, the wells were washed three times by wash buffer. Subsequently, adding Standard, sample, or control per well and incubating for 2 hours at room temperature. Again, wells were washed for three times and added with conjugate for 1 hours at room temperature. Then added substrate solution to each well and incubrated for 20 minutes. Stop solution was added to wells and optical density 450 nm of each well was measured by a microplate reader.
