# Supplementary material for: Downregulation of ASPP2 promotes gallbladder cancer metastasis and macrophage recruitment via aPKC-ι/GLI1 pathway
Source: Cell Death Dis. 2018 Nov 2;9(11):1115. doi: 10.1038/s41419-018-1145-1 (PMC6214900; doi:10.1038/s41419-018-1145-1)
Supplement: Supplementary file 2 — Supplemental Figure Legends [file 41419_2018_1145_MOESM2_ESM.docx]

**Supplemental Figure Legends**

**Figure S1. ASPP2 involved in cell adhesion and tight junctions in Hepatocellular carcinoma and Cholangiocarcinoma.** (A-D) GSEA results revealed significant enrichment of cell adhesion and tight junction target genes in Hepatocellular carcinoma and Cholangiocarcinoma.

**Figure S2. ASPP2 deficiency promoted the proliferation and induced EMT of GBC cells.** (A) Western blotting was performed to detect the expression of ASPP2 in GBC-SD, OCUG-1 and NOZ cells. (B) Immunofluorescence analysis showing the expression levels of ASPP2, E-cadherin, N-cadherin, and vimentin in NOZ and OCUG-1 cells with lentivirus-mediated ASPP2 knockdown (KD) or overexpression (ASPP2). A lentiviral vector containing an siRNA that did not recognize any human gene was constructed as a negative control (NC). Cells without any treatment were used as the blank control (Blank). Scale bar, 50 μm. (C-D) Western blotting and qPCR were performed to detect the expression levels of ZEB1 and β-catenin in NOZ and OCUG-1 cells with the indicated treatment. (E) Bar graph for showing the weight of node mouse after treated with indicated GBC cells. (F) IHC was used to detect ASPP2 expression in xenograft tumor. Representative images were shown. Scale bar, 50 μm. Data are from three independent experiments and presented as means ± SDs.

**Figure S3.** **ASPP2 KD promoted the recruitment of macrophages and enhanced the secretion of cytokine.** (A) qPCR was used to analyze the expression of cytokines in the indicated GBC cells. (B) IHC staining showing CD163 expression in xenograft tumors after treatment with the indicated cells lines. Scale bar, 50 μm. (C-E) ELSIA for analyzing the level of CCL2, CCL5 and TNF-α in the supernatant of GBC cells with indicated treatment. **P*<0.05, ***P*<0.01. Data are derived from three independent experiments and presented as means ± SDs.

**Figure S4. Depletion of ASPP2 regulated GLI1 transcriptional activity via noncanonical Hh signaling.** (A-B) qPCR and western blotting were performed to detect the expression levels of PTCH1 and SMO in GBC cells with the indicated treatment. (C) qPCR was used to analyze the mRNA expression of GLI1 in GBC cells treated with cyclopamine. (D) Western blotting was used to analyze the protein expression of SMO in the indicated GBC cells. (E) The distributions of GLI1 and ASPP2 were detected by nuclear and cytoplasmic fraction in NOZ cells. (F) IHC staining showing the ASPP2-induced nuclear accumulation of GLI1 in xenograft tumors. Scale bar, 50 μm. **P*<0.05, ***P*<0.01. Data are derived from three independent experiments and presented as means ± SDs.

**Figure S5. ASPP2 expression was inversely correlated with GLI1 expression and TAMs infiltration in human GBC.** (A-B) IHC staining showing the expression of ASPP2, GLI1, CD68 and CD163 in representative GBC tissues and pair-matched normal tissues. Scale bar, 50 μm.
