# Supplementary figures and images for: Downregulation of ASPP2 promotes gallbladder cancer metastasis and macrophage recruitment via aPKC-ι/GLI1 pathway
Source: Cell Death Dis. 2018 Nov 2;9(11):1115. doi: 10.1038/s41419-018-1145-1 (PMC6214900; doi:10.1038/s41419-018-1145-1)

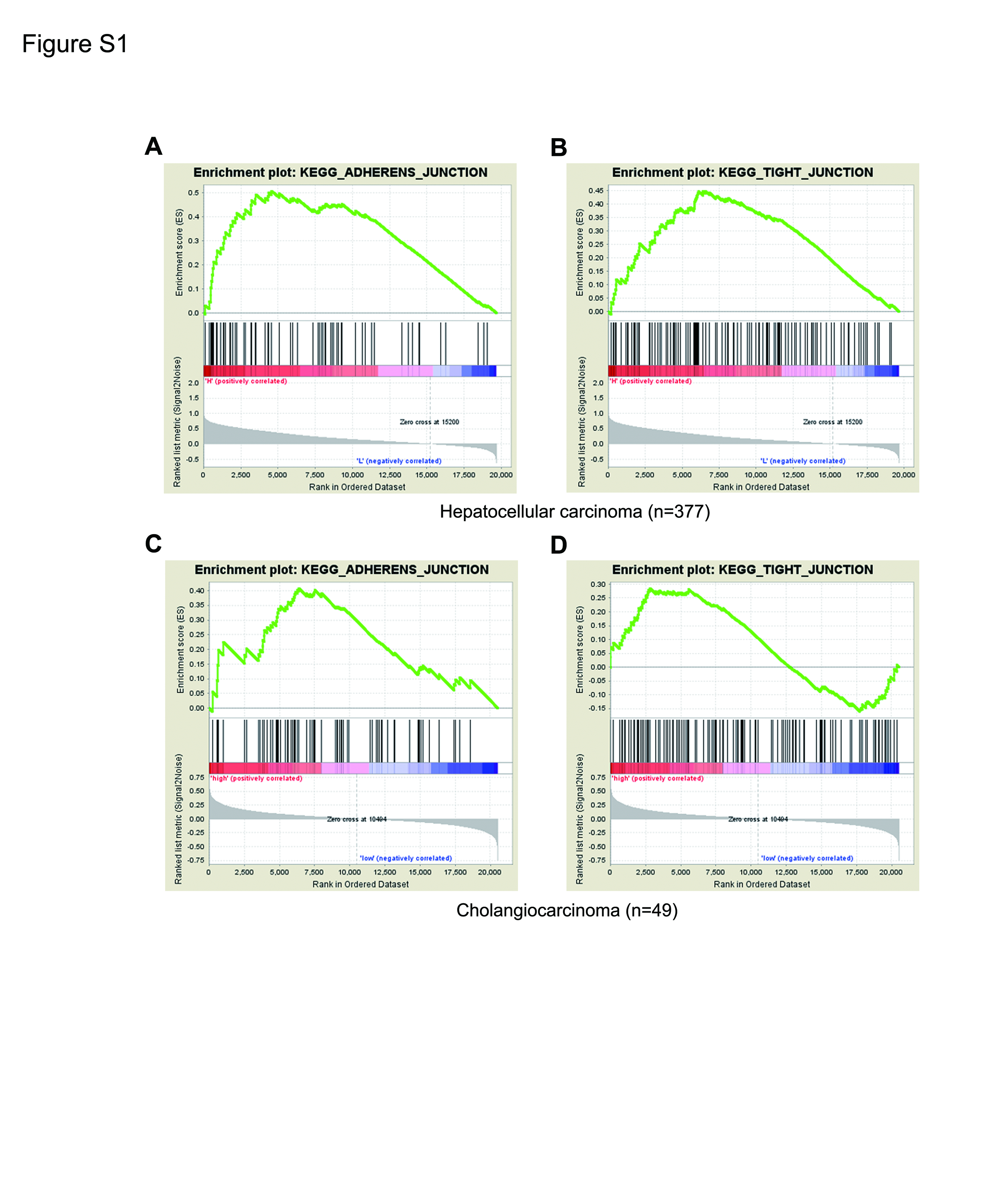

Supplement: Supplementary file 3 — Figure S1 [file 41419_2018_1145_MOESM3_ESM.tif]

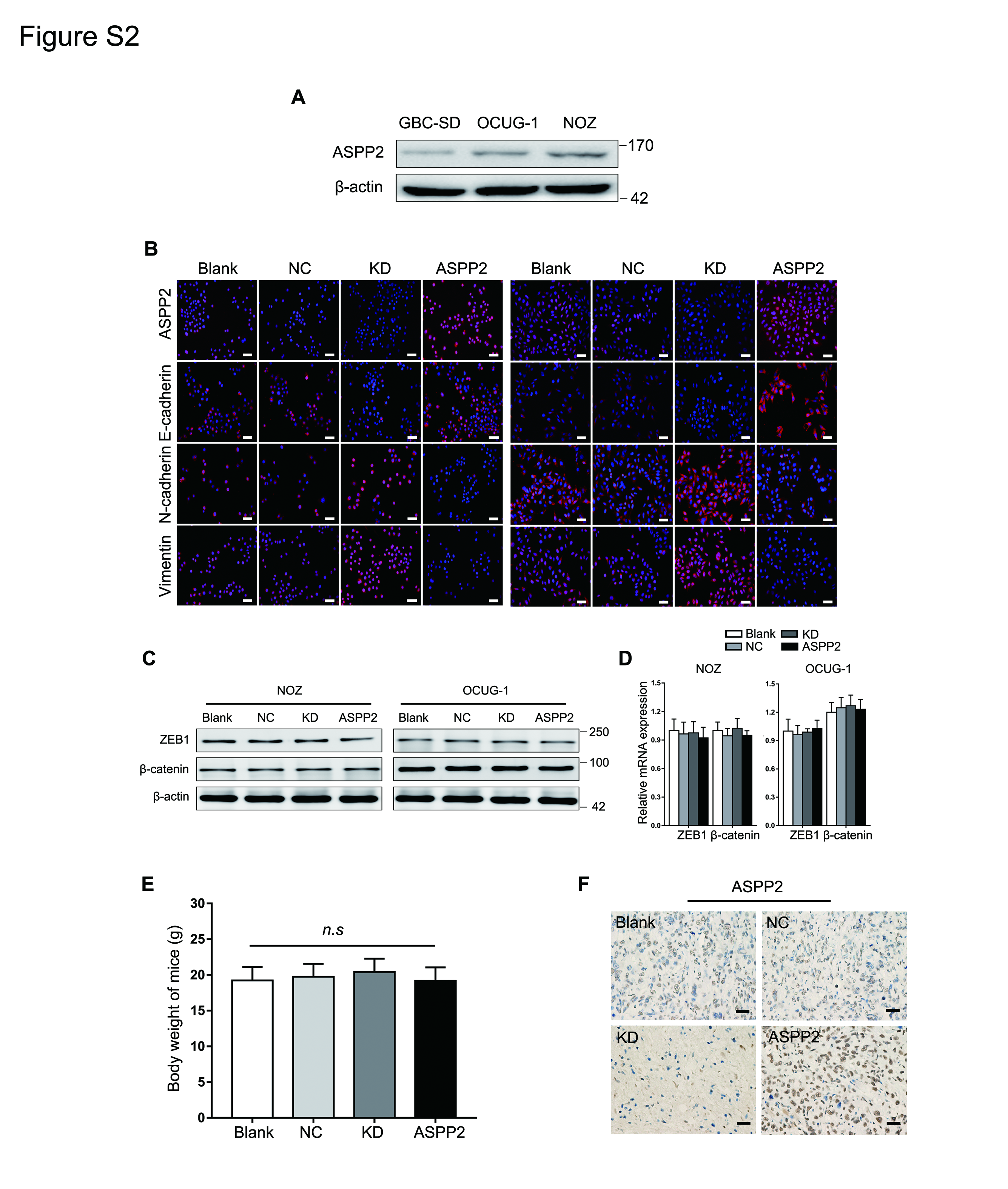

Supplement: Supplementary file 4 — Figure S2 [file 41419_2018_1145_MOESM4_ESM.tif]

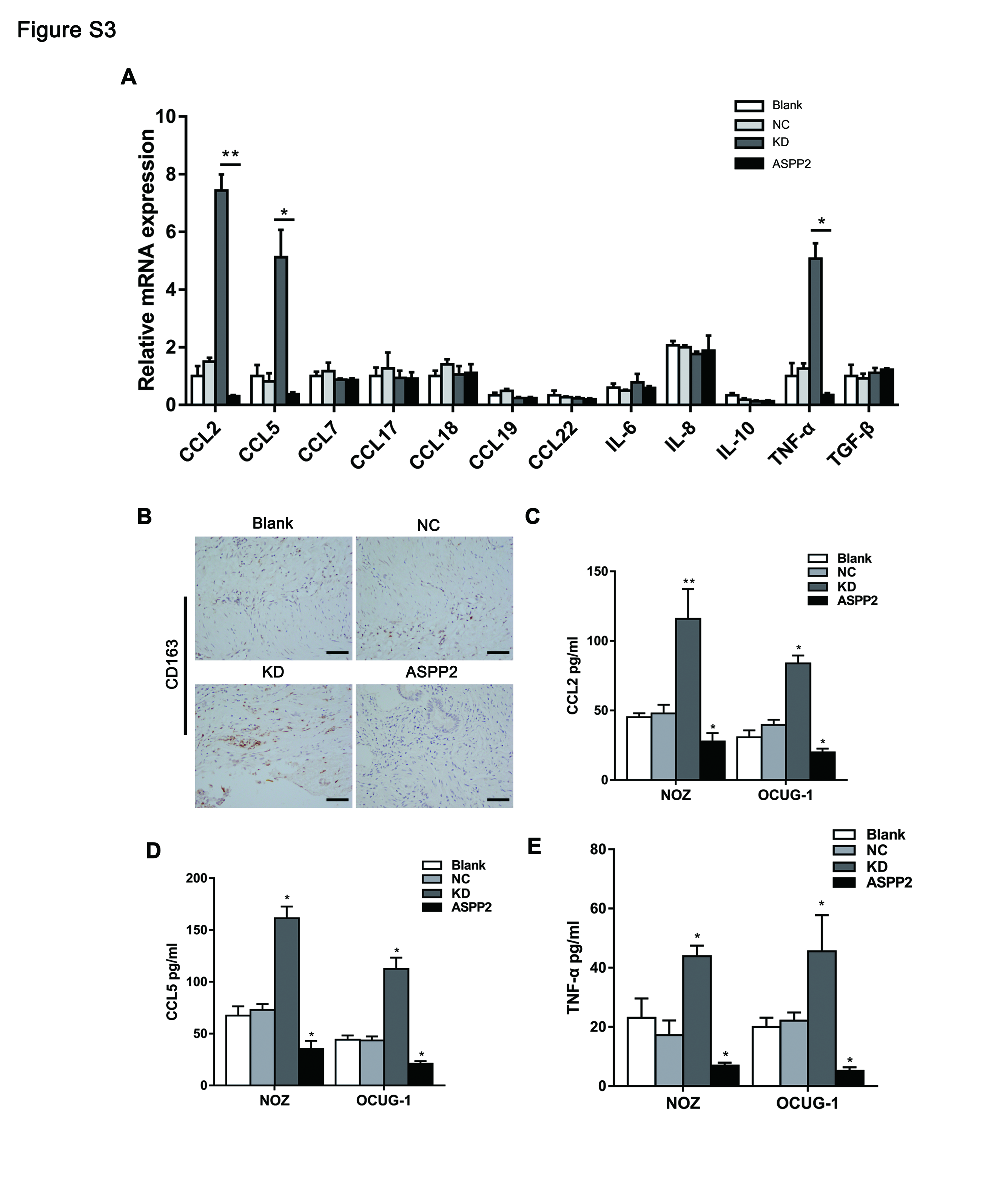

Supplement: Supplementary file 5 — Figure S3 [file 41419_2018_1145_MOESM5_ESM.tif]

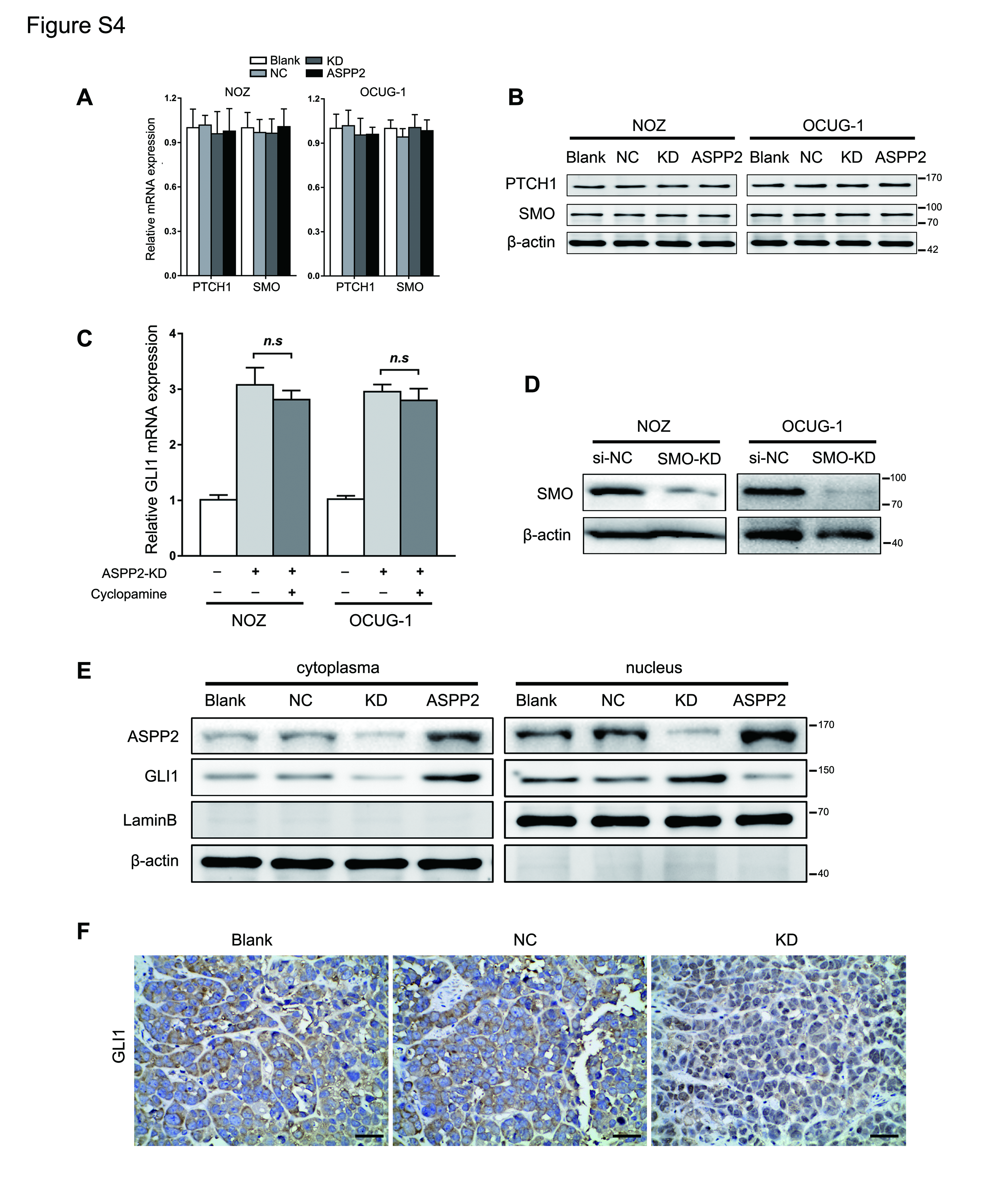

Supplement: Supplementary file 6 — Figure S4 [file 41419_2018_1145_MOESM6_ESM.tif]

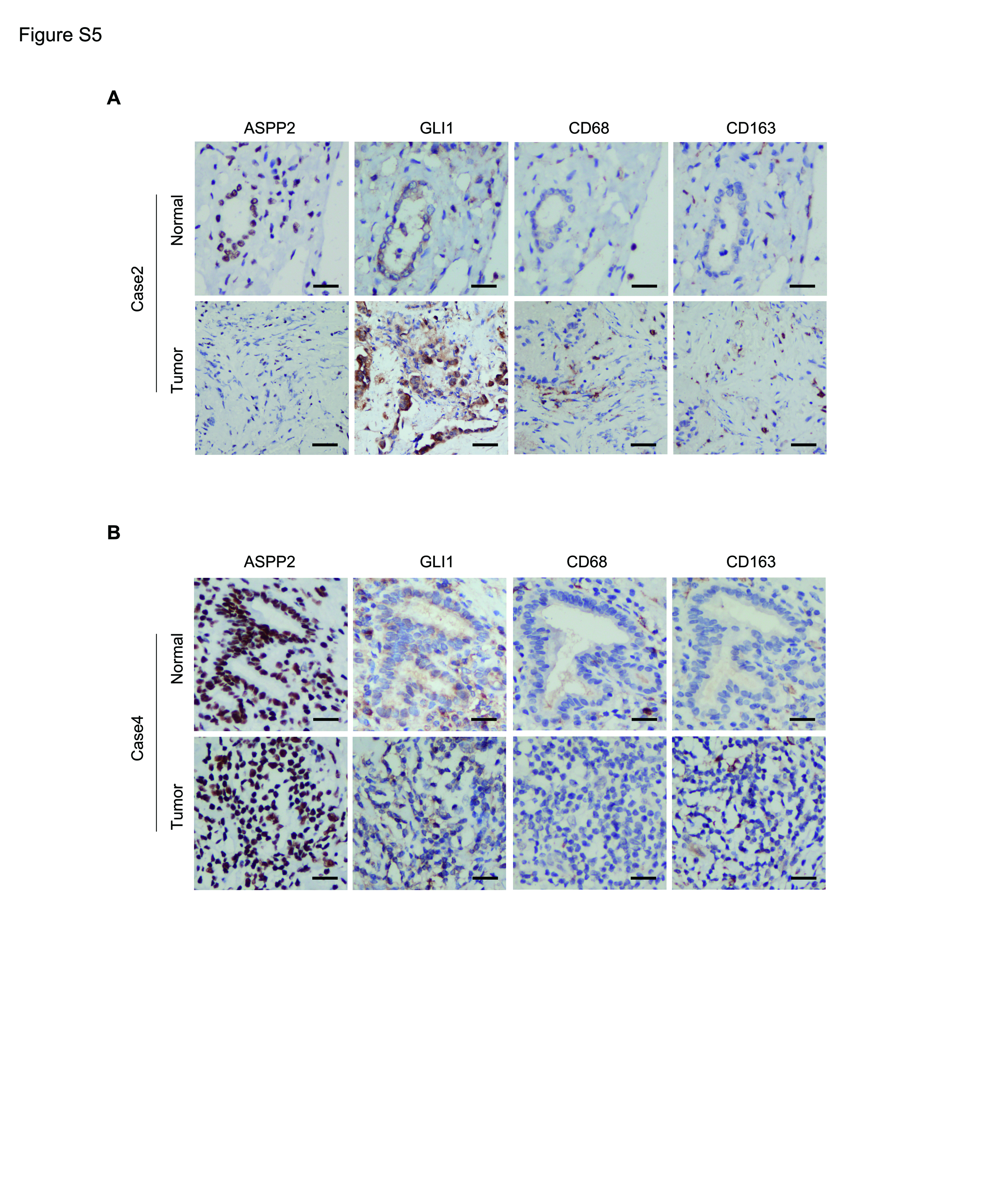

Supplement: Supplementary file 7 — Figure S5 [file 41419_2018_1145_MOESM7_ESM.tif]
