# Supplementary material for: Downregulation of ASPP2 promotes gallbladder cancer metastasis and macrophage recruitment via aPKC-ι/GLI1 pathway
Source: Cell Death Dis. 2018 Nov 2;9(11):1115. doi: 10.1038/s41419-018-1145-1 (PMC6214900; doi:10.1038/s41419-018-1145-1)
Supplement: Supplementary file 8 — Supplementary tables [file 41419_2018_1145_MOESM8_ESM.docx]

**Supporting Table S1. Clinicopathological characteristics of 72 GBC patients**

| **Characteristic** | **Gallbladder cancer (n=72)** | |
| --- | --- | --- |
|  | **number** | **percentage** |
| Age |  |  |
| <60 | 39 | 54.17 |
| ≥60 | 33 | 45.83 |
| Gender |  |  |
| Male | 20 | 27.78 |
| Female | 52 | 72.22 |
| Lymph node metastasis |  |  |
| Present | 41 | 56.94 |
| Absent | 31 | 43.05 |
| TNM stage |  |  |
| I/II | 24 | 33.33 |
| III/IV | 48 | 66.67 |
| Differentiation |  |  |
| Well | 18 | 25.00 |
| Moderate/poor | 54 | 75.00 |

**Supporting Table S2. Correlation between aPKC-ι, GLI1 and clinicopathological characteristics in GBC patients**

| **Characteristic** | **No.** | **aPKC-ι** | | ***P*** | **GLI1** | | ***P*** |
| --- | --- | --- | --- | --- | --- | --- | --- |
|  |  | Low | High |  | Low | High |  |
| Age |  |  |  | 0.612 |  |  | 0.686 |
| <60 | 39 | 14 | 25 |  | 16 | 23 |  |
| ≥60 | 33 | 9 | 24 |  | 12 | 21 |  |
| Gender |  |  |  | 0.730 |  |  | 0.509 |
| Male | 20 | 7 | 13 |  | 9 | 11 |  |
| Female | 52 | 16 | 36 |  | 19 | 33 |  |
| Tumor/non-tumor tissue |  |  |  | **<0.001** |  |  | **0.002** |
| Tumor | 72 | 23 | 49 |  | 28 | 44 |  |
| Non-tumor | 72 | 51 | 21 |  | 47 | 25 |  |
| Lymph node metastasis |  |  |  | **<0.001** |  |  | **<0.001** |
| Present | 41 | 6 | 35 |  | 8 | 33 |  |
| Absent | 31 | 17 | 14 |  | 20 | 11 |  |
| TNM stage |  |  |  | **<0.001** |  |  | **<0.001** |
| I/II | 24 | 16 | 8 |  | 17 | 7 |  |
| III/IV | 48 | 7 | 41 |  | 11 | 37 |  |
| Differentiation |  |  |  | **<0.001** |  |  | **<0.001** |
| Well | 18 | 8 | 3 |  | 14 | 4 |  |
| Moderate/poor | 54 | 15 | 46 |  | 14 | 40 |  |

**Supporting Table S3. Univariate and multivariate analyses for predictors of overall survival**

| **Vriables** | **Overall survival** | | | | | |
| --- | --- | --- | --- | --- | --- | --- |
|  | **Univariate analysis** | | | **Multivariate analysis** | | |
|  | **HR** | **95%CI** | ***P* value** | **HR** | **95%CI** | ***P* value** |
| Age(<60 vs ≥60) | 1.014 | 0.614-1.674 | 0.957 |  |  |  |
| Gender(male vs female) | 0.822 | 0.473-1.429 | 0.487 |  |  |  |
| Lymph node metastasis(no vs yes) | 10.653 | 5.109-22.211 | <0.001 | 2.924 | 1.134-7.545 | 0.026 |
| TNM stage (I/II vs III/IV) | 23.319 | 8.426-64.533 | <0.001 | 9.892 | 2.067-47.328 | 0.004 |
| Differentiation (well vs moderate/poor) | 9.202 | 3.996-21.189 | <0.001 | 3.232 | 1.049-9.957 | 0.041 |
| ASPP2 expression (low vs high) | 0.087 | 0.038-0.200 | <0.001 | 0.183 | 0.059-0.564 | 0.003 |
| aPKC-ι expression (low vs high) | 5.098 | 2.664-9.756 | <0.001 | 2.333 | 1.015-5.364 | 0.046 |
| GLI1 expression (low vs high) | 6.050 | 3.132-11.686 | <0.001 | 5.210 | 2.405-11.286 | <0.001 |

**Supporting Table S4. Information of antibodies or drugs used in this study**

| **Antibody/Drug** | **Company** | **Cat.Number** | **Use** |
| --- | --- | --- | --- |
| ASPP2 | Abcam | ab181377 | WB 1:10000 |
|  |  |  | IHC 1:200 |
|  |  |  | IF 1:50 |
|  |  | ab70548 | IP 2 µg/IP |
| E-cadherin | ProteinTech Group | 20874-1-AP | WB 1:1000 |
|  |  |  | IF 1:200 |
| N-cadherin | Cell Signaling Technology | #14215 | WB 1:1000 |
|  |  |  | IF 1:200 |
| Vimentin | ProteinTech Group | 10366-1-AP | WB 1:1000 |
|  |  |  | IF 1:250 |
| aPKC-ι | ProteinTech Group | 13883-1-AP | WB 1:1000 |
|  |  |  | IHC 1:200 |
|  |  |  | IP 2 µg/IP |
| GLI1 | Cell Signaling Technology | #2534 | WB 1:1000 |
|  |  |  | IP 1:50 |
|  | Boster | PB0142 | IHC 1:200 |
| p-GLI1 |  |  |  |
| CD68 | ProteinTech Group | 25747-1-AP | WB 1:800 |
|  |  |  | IHC 1:200 |
| CD163 | Abcam | ab87099 | WB 1:800 |
|  |  |  | IHC 1:200 |
| F4/80 | Biolegend | 123109 | FC |
| CD11b^+^ | Biolegend | 101211 | FC |
| CCL2 | Cell Signaling Technology | #2027 | WB 1:1000 |
|  | Invitrogen | BMS281 | ELSIA |
| CCL5 | Boster | PB0653 | WB 1:800 |
|  | Abcam | ab174446 | ELSIA |
| TNF-α | Cell Signaling Technology | # 3707S | WB 1:1000 |
| TNF-α | R&D Systems | P01375 | ELSIA |
| PTCH1 | ProteinTech Group | 17520-1-AP | WB 1:800 |
| SMO | Abcam | ab72130 | WB 1:5000 |
| ZEB1 | ProteinTech Group ProteinTech Group | 21544-1-AP | WB 1:1000 |
| Beta-Catenin | ProteinTech Group | 51067-2-AP | WB 1:1000 |
| Snai1 | ProteinTech Group | 13099-1-AP | WB 1:1000 |
| LaminB | Santa Cruz Biotechnology | sc-56144 | WB 1:800 |
| β-actin | Abcam | ab8227 | WB 1:5000 |
| Cyclopamine | Selleck | S1146 | 5μM |
| PSI | MedChemexpress | HY-13689 | 10μM |

**Supporting Table S5. Information of primers and target sequences used in this study**

| **qPCR** | **Forward (5’ to 3’)** | **Reverse (5’ to 3’)** | **Amplicon** |
| --- | --- | --- | --- |
| ASPP2 | AAGCAATGGGAAACTTGTGG | GCATCTCTAGCTGCCTGGTC | 119 |
| E-cadherin | CTGGACAGGGAGGATTTTGA | ACCTGAGGCTTTGGATTCCT | 191 |
| N-cadherin | CGTGAAGGTTTGCCAGTGT | GCACAAGGATAAGCAGGATGA | 128 |
| Vimentin | AGAGAACTTTGCCGTTGAAGC | ACGAAGGTGACGAGCCATT | 100 |
| ZEB1 | CAGGCAAAGTAAATATCCCTGC | GGTAAAACTGGGGAGTTAGTCA | 124 |
| β-catenin | TGGATTGATTCGAAATCTTGCC | GAACAAGCAACTGAACTAGTCG | 92 |
| CCL2 | GCCTCCAGCATGAAAGTCTC | AGGTGACTGGGGCATTGAT | 109 |
| CCL5 | TACACCAGTGGCAAGTGCTC | TGTACTCCCGAACCCATTTC | 100 |
| CCL7 | GAAAGCCTCTGCAGCACTTC | TAGCTCTCCAGCCTCTGCTT | 150 |
| CCL17 | AGGGACCTGCACACAGAGAC | CTCGAGCTGCGTGGATGTGC | 133 |
| CCL18 | CTCTGCTGCCTCGTCTATACCT | CTTGGTTAGGAGGATGACACCT | 108 |
| CCL19 | CCTGCTGGTTCTCTGGACTT | CTCACGATGTACCCAGGGAT | 108 |
| CCL22 | ATGGCTCGCCTACAGACTGCACTC | CACGGCAGCAGACGCTGTCTTCCA | 114 |
| IL-6 | AATAACCACCCCTGACCCAAC | ACATTTGCCGAAGAGCCCT | 149 |
| IL-8 | AAACCACCGGAAGGAACCAT | CCTTCACACAGAGCTGCAGAAA | 101 |
| IL-10 | AACAAGAGCAAGGCCGTGG | GAAGATGTCAAACTCACTCATGGC | 93 |
| TNF-α | TCAGCCTCTTCTCCTTCCTG | GCCAGAGGGCTGATTAGAGA | 124 |
| TGF-β | AAGGACCTCGGCTGGAAGTGC | CCGGGTTATGCTGGTTGTA | 137 |
| aPKC-ι | TACGGCCAGGAGATACAACC | CATCTGGAGTGAGCTGGACA | 489 |
| GLI1 | GGCAGCACTGAAGACCTCTC | ATTGGCCGGAGTTGATGTAG | 119 |
| PTCH1 | GTCCTGCTGGTTGCACTGTC | TCCACACCAACACCAAGAGC | 119 |
| SMO | GAGCCCACCTCCAATGAGAC | GGGCTTTGAAGGAAGTGTGC | 124 |
| YAP1 | CACAGCATGTTCGAGCTCAT | CTGAGCTGTGGGTGTAGCTG | 116 |
| CTGF | GTTCCAAGACCTGTGGGATG | TCTCTTCCAGGTCAGCTTCG | 120 |
| CYR61 | CCCAGTGCTCAAAGACCTGT | TACACTGGCTGTCCACAAGG | 121 |
| ANKRD | TGAAGGCTGCTCTGGAGAAT | CCAAATGTCCTTCCAAGCAT | 126 |
| β-actin | GTCCACCGCAAATGCTTCTA | TGCTGTCACCTTCACCGTTC | 190 |

| **ChIP** | **Forward (5’ to 3’)** | **Reverse (5’ to 3’)** | **Amplicon** |
| --- | --- | --- | --- |
| aPKC-ι | CCACTTGCAGGAATGACAGCG | GCGAAGACGAACTCTAGGGTCCAGT | 174 |
| CCL2 | ATTCCCGAAATACTCCTCCAC | TGGTTATGGCAGCTATTCTCC | 80 |
| CCL5 | GGAAAAAATGATCCCTAAAGTCC | GCTTATCTGTGCCAGCCACTA | 184 |
| TNFA | AGAGGGGAATAATAGAAGAACATC | GTGATCCCTCATGCCTACTTC | 118 |

| **Gene** | **Target sequence** |
| --- | --- |
| ASPP2 | GGACTGTACCCAAGAATTA |
| GLI1 | CTCCACAGGCATACAGGAT |
| aPKC-ι | AGTCTAGGTCTTCAGGATT |
