# Supplementary material for: Downregulation of ASPP2 promotes gallbladder cancer metastasis and macrophage recruitment via aPKC-ι/GLI1 pathway
Source: Cell Death Dis. 2018 Nov 2;9(11):1115. doi: 10.1038/s41419-018-1145-1 (PMC6214900; doi:10.1038/s41419-018-1145-1)
Supplement: Supplementary file 9 — The STR authentication of GBC cell lines [file 41419_2018_1145_MOESM9_ESM.pdf]

GENEWIZ®

Solid science. Superior service.

Cell Line Authentication Report

GENEWIZ,Inc. Beijing

30 Science Park Road

Zhong-Guan-Cun Life Science Park

Changping District, 102206

Beijing, China

Tel: 400-8100-669

Fax: 010-59458058

Email: Genomics.China@genewiz.com.cn

www.genewiz.com.cn

GENEWIZ,Inc. Beijing
30 Life Science Park Road
Changping District, 102206
Beijing, China
Tel: 400-8100-669
Fax: 010-59458058
www.genewiz.com.cn

Cell Line Authentication Report

Customer: JiangLin

Institution: Xin Hua Hospital Affiliated to ShangHai Jiao Tong University School of Medicine

Quotation Number: HJ1601121

Completion Date： 02/04/2016

1. **Sample ID: NOZ**
2. **Original Material: Cell pellets**
3. **Methods:**

1).Genomic DNA was extracted from the cell pellets provided by the customer.

2).Samples, together with positive and negative control were amplified using GenePrint 10 System(Promega).

3). Amplified products were processed using the ABI3730xl Genetic Analyzer.

4).Data were analyzed using GeneMapper4.0 software and then compared with the ATCC, DSMZ or JCRB databases for reference matching.

4. **Results:**

1) 10 Loci STR Profile：

Genetic Site	ATCC		Customer sample	
(Locus)	NOZ		NOZ	
Amelogenin	X		X	
CSF1PO	11	13	11	13
D13S317	8	12	8	12
D16S539	9	11	9	11
D5S818	11	13	11	13
D7S820	10	11	10	11
THO1	7	9	7	9
TPOX	8	11	8	11
vWA	19		19	
D21S11			30	31
Percent match between the sample and the database				

GENEWIZ,Inc. Beijing
30 Life Science Park Road
Changping District, 102206
Beijing, China
Tel: 400-8100-669
Fax: 010-59458058
www.genewiz.com.cn

profile：100%

Summary:

1) Your cell line is considered to be “identical” tothe reference cell line in the ATCC STR database, as the STR profile yields a 100% match.

Notes：

1. P=100% x (2xM)/N; M=16, N=32 P=100% x (2x16)/32=100%

M: number of the matching peaks; N: number of all peaks

2. Based on the ANSI Standard, cell lines with ≥80% match are considered to be related; i.e.,derived from a common ancestry. Cell lines with between a 55% to 80% match require futher profiling for authentication of relatedness.

3. The short tandem repeat (STR) profile generated by GENEWIZ Inc. is indicative only of the sample sent to GENEWIZ Inc. at the time it was sent. This data and analysis are for research use only.

2) Electrophoretogram

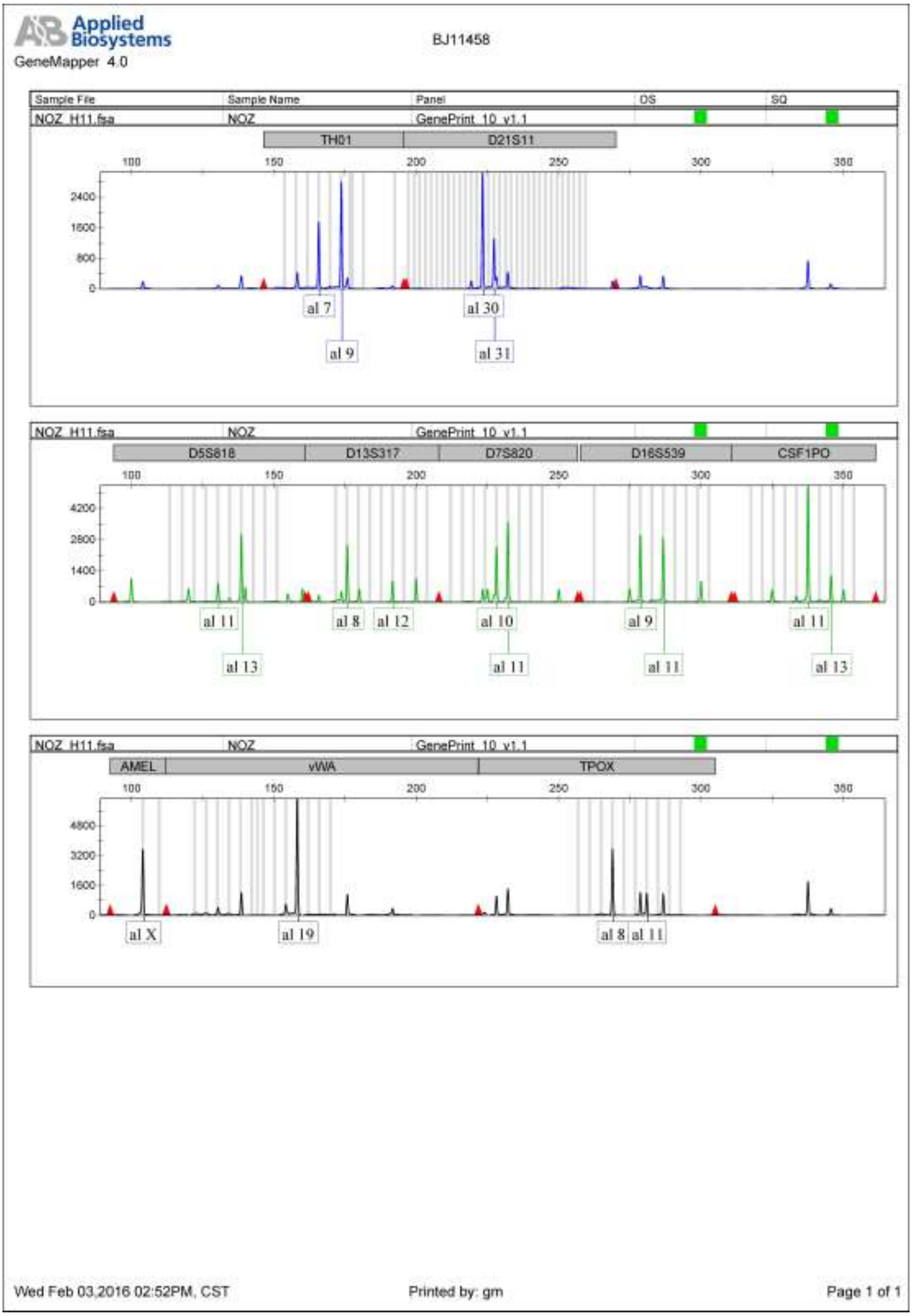

Note：Raw data in appendix

GENEWIZ®

Solid science. Superior service.

Cell Line Authentication Report

GENEWIZ,Inc. Beijing

30 Science Park Road

Zhong-Guan-Cun Life Science Park

Changping District, 102206

Beijing, China

Tel: 400-8100-669

Fax: 010-59458058

Email: Genomics.China@genewiz.com.cn

www.genewiz.com.cn

GENEWIZ, Solid science. Superior service.

GENEWIZ,Inc. Beijing
30 Life Science Park Road
Changping District, 102206
Beijing, China
Tel: 400-8100-669
Fax: 010-59458058
www.genewiz.com.cn

Cell Line Authentication Report

Customer: JiangLin

Institution: Xin Hua Hospital Affiliated to Shanghai Jiao Tong University School of Medicine

Quotation Number: GWBJYJ15121801

Completion Date：12/30/2015

1. **Sample ID: OCUG-1**
2. **Original Material: Cell pellets**
3. **Methods:**

1).Genomic DNA was extracted from the cell pellets provided by the customer.

2).Samples, together with positive and negative control were amplified using GenePrint 10 System(Promega).

3). Amplified products were processed using the ABI3730xl Genetic Analyzer.

4).Data were analyzed using GeneMapper4.0 software and then compared with the ATCC, DSMZ or JCRB databases for reference matching.

4. **Results:**

1) **10 Loci STR Profile：**

Genetic Site	JCRB		Customer sample	
(Locus)	OCUG-1		OCUG-1	
Amelogenin	X		X	
CSF1PO	10	12	10	12
D13S317	8	11	8	11
D16S539	9	13	9	13
D5S818	10	12	10	12
D7S820	10	11	10	11
THO1	7	？	7	9.3
TPOX	8	11	8	11
vWA	？ 15	16	16	
D21S11			28	29
Percent match between the sample and the database				

GENEWIZ,Inc. Beijing
30 Life Science Park Road
Changping District, 102206
Beijing, China
Tel: 400-8100-669
Fax: 010-59458058
www.genewiz.com.cn

profile：88.2%

Summary:

1) Your cell line is considered “related”to the reference cell line in the ATCC STR database, as the STR profile yields matches that are ≥ 80% but less than 100%.

Notes：

1. P=100% x (2xM)/N; M=15, N=34 P=100% x (2x15)/34=88.2%

M: number of the matching peaks; N: number of all peaks

2. Based on the ANSI Standard, cell lines with ≥80% match are considered to be related; i.e.,derived from a common ancestry. Cell lines with between a 55% to 80% match require futher profiling for authentication of relatedness.

3. The short tandem repeat (STR) profile generated by GENEWIZ Inc. is indicative only of the sample sent to GENEWIZ Inc. at the time it was sent. This data and analysis are for research use only.

2) Electrophoretogram

GENEWIZ
Solid science. Superior service.

GENEWIZ,Inc. Beijing
30 Life Science Park Road
Changping District, 102206
Beijing, China
Tel: 400-8100-669
Fax: 010-59458058
www.genewiz.com.cn

Applied Biosystems
GeneMapper 4.0

BJ11387

Sample File	Sample Name	Panel	OS	SQ
OCUG-1_D12.fsa	OCUG-1	GenePrint_10_v1.1		

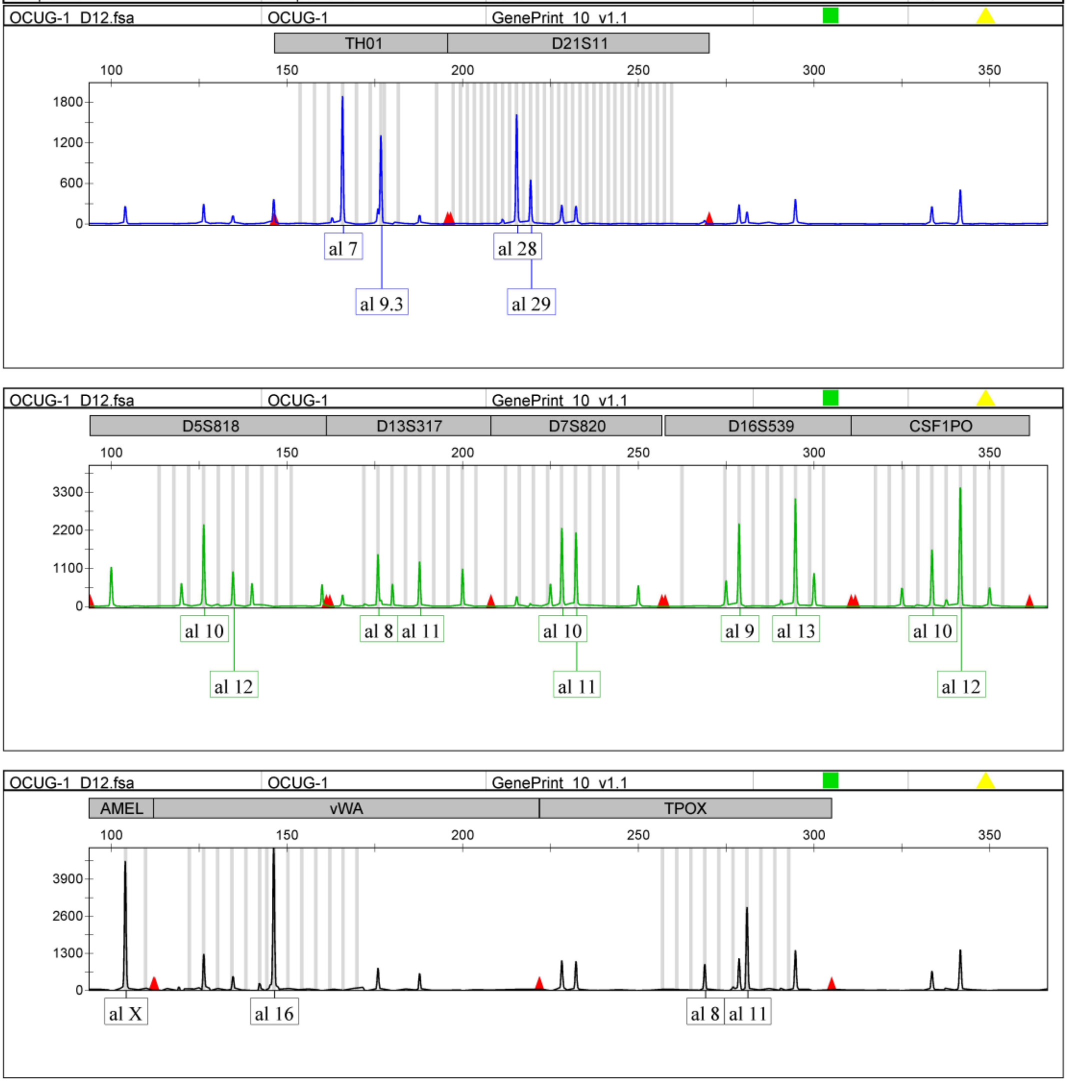

Wed Dec 30,2015 03:13PM, CST   Printed by: gm   Page 1 of 1

Note：Raw data in appendix
